# Multi-view fusion of diffusion MRI microstructural models: a preterm birth study

**DOI:** 10.3389/fnins.2024.1480735

**Published:** 2024-12-20

**Authors:** Rosella Trò, Monica Roascio, Domenico Tortora, Mariasavina Severino, Andrea Rossi, Eleftherios Garyfallidis, Gabriele Arnulfo, Marco Massimo Fato, Shreyas Fadnavis

**Affiliations:** ^1^Department of Informatics, Bioengineering, Robotics and System Engineering (DIBRIS), University of Genoa, Genoa, Italy; ^2^Neuroradiology Unit, IRCCS Istituto Giannina Gaslini, Genoa, Italy; ^3^Department of Health Sciences (DISSAL), University of Genoa, Genoa, Italy; ^4^Intelligent Systems Engineering, Indiana University Bloomington, Bloomington, IN, United States; ^5^Neuroscience Center, Helsinki Institute of Life Science, University of Helsinki, Helsinki, Finland; ^6^Massachusetts General Hospital, Harvard Medical School, Boston, MA, United States

**Keywords:** diffusion Magnetic Resonance Imaging, preterm birth, intramodal imaging approach, inference, prediction

## Abstract

**Objective:**

High Angular Resolution Diffusion Imaging (HARDI) models have emerged as a valuable tool for investigating microstructure with a higher degree of detail than standard diffusion Magnetic Resonance Imaging (dMRI). In this study, we explored the potential of multiple advanced microstructural diffusion models for investigating preterm birth in order to identify non-invasive markers of altered white matter development.

**Approach:**

Rather than focusing on a single MRI modality, we studied on a compound of HARDI techniques in 46 preterm babies studied on a 3T scanner at term-equivalent age and in 23 control neonates born at term. Furthermore, we investigated discriminative patterns of preterm birth using multiple analysis methods, drawn from two only seemingly divergent modeling goals, namely inference and prediction. We thus resorted to (i) a traditional univariate voxel-wise inferential method, as the Tract-Based Spatial Statistics (TBSS) approach; (ii) a univariate predictive approach, as the Support Vector Machine (SVM) classification; and (iii) a multivariate predictive Canonical Correlation Analysis (CCA).

**Main results:**

The TBSS analysis revealed significant differences between preterm and term cohorts in several white matter areas for multiple HARDI features. SVM classification on skeletonized HARDI measures yielded satisfactory accuracy, particularly for highly informative parameters about fiber directionality. Assessment of the degree of overlap between the two methods in voting for the most discriminating features exhibited a good, though parameter-dependent, rate of agreement. Finally, CCA identified joint changes precisely for those measures exhibiting less correspondence between TBSS and SVM.

**Significance:**

Our results suggest that a data-driven intramodal imaging approach is crucial for gathering deep and complementary information. The main contribution of this methodological outline is to thoroughly investigate prematurity-related white matter changes through different inquiry focuses, with a view to addressing this issue, both aiming toward mechanistic insight and optimizing predictive accuracy.

## 1 Introduction

Diffusion Magnetic Resonance Imaging (dMRI) has established itself as a cornerstone in the study of brain microstructure, offering unmatched sensitivity for non-invasive imaging compared to conventional MRI (Basser et al., 1994; Le Bihan et al., 2001). The advent of High Angular Resolution Diffusion Imaging (HARDI) (Descoteaux, 1999) has propelled dMRI into a new era of precision, enabling the exploration of microstructural features beyond the capabilities of traditional Diffusion Tensor Imaging (DTI) (Tournier et al., 2011). By providing deeper insights into cellular architecture, HARDI-based approaches are particularly valuable for studying white matter (WM) development in complex scenarios, such as preterm birth, where structural abnormalities can be subtle but widespread.

Despite advances in neonatal care, preterm birth remains a global challenge (Beck et al., 2010; Blencowe et al., 2013). Approximately 50% of survivors experience long-term neurodevelopmental impairments, including cognitive, motor, and behavioral difficulties (Bhutta et al., 2002). These outcomes are often associated with disruptions in WM integrity, which can hinder neuronal connectivity and delay brain maturation (Kimpton et al., 2021; Dyet et al., 2006; Tortora et al., 2018). Understanding the nature and extent of these disruptions is critical for improving diagnostics and informing targeted interventions.

Recent developments in dMRI have significantly improved our understanding of preterm brain development and injury, providing non-invasive insights into WM microstructure (Pannek et al., 2014). Studies indicate that preterm birth often leads to disruptions in cortical microstructure and neuronal connectivity, contributing to developmental disabilities (Dudink et al., 2015). While cystic periventricular WM damage has been linked to abnormal motor development, the relationship between diffuse WM damage and long-term developmental outcomes remains unclear (Hart et al., 2008). Advanced dMRI techniques have revealed alterations in brain region size, volume, and growth rates following preterm birth, with these changes correlated with diminished motor, cognitive, and behavioral performance from childhood into adulthood (Pandit et al., 2013; Volpe, 2003; Counsell et al., 2003; Zhao et al., 2021; Ouyang et al., 2019b; Shi et al., 2016; Kelly et al., 2016; Pannek et al., 2018). As these imaging techniques evolve, they possess potential as biomarkers for predicting outcomes and evaluating interventions in preterm infants.

To unlock the broader biological implications of these findings, it is essential to integrate diverse dMRI models and innovative analytical frameworks. Based on this need, our study employs several advanced HARDI-based diffusion models to investigate preterm-related WM abnormalities comprehensively. Specifically, we focus on a variety of models that have been selected for their suitability in capturing microstructural changes beyond DTI's capabilities (Pecheva et al., 2018). These models include Diffusion Kurtosis Imaging (DKI) (Jensen et al., 2005), Neurite Orientation Dispersion and Density Imaging (NODDI) (Zhang et al., 2012), Multi-Shell Multi-Tissue Constrained Spherical Deconvolution (MSMT-CSD) (Jeurissen et al., 2014), and FORECAST (Anderson, 2005; Kaden et al., 2016), to capture a more nuanced understanding of the WM changes associated with prematurity.

To explore these microstructural alterations, we adopt a dual analytical framework combining inference and prediction. Indeed, as stated in Bzdok and Ioannidis (2019) and Bzdok et al. (2020), in the case of complex biological systems, such as the human brain, resorting to these two seemingly diverging modeling goals provides a better understanding of their complex interactions. The objective of inference entails prioritizing the contribution of each input variable through null hypothesis significance testing. In contrast, the predictive regime emphasizes on the relevance of the output of the model for accurate forecasting.

In this study, we employ two state-of-the-art univariate techniques representing these analytical paradigms: Tract-Based Spatial Statistics (TBSS) and Support Vector Machines (SVM). TBSS is a voxel-wise inferential method designed to detect statistically significant differences in WM microstructure across cohorts. It is widely recognized for its robustness and observer-independent nature, making it an effective tool for group-level analysis. However, its limitations in detecting diffuse abnormalities and providing personalized metrics highlight the need for complementary approaches (Smith et al., 2006). SVM, in contrast, represents a predictive, data-driven method that offers individualized classification capabilities. By uncovering discriminatory patterns between preterm and term cohorts, SVM bridges the gap between group-level analyses and clinical applications such as early diagnosis and prognosis (Golland et al., 2002; Lao et al., 2004).

Finally, to address potential redundancies in dMRI models and uncover biologically interpretable components, we also move beyond univariate analysis methods, summarizing single microstructural features at a time, toward a multivariate predictive model via Canonical Correlation Analysis (CCA) (Hardoon et al., 2004). This method integrates multiple diffusion metrics to reveal shared and distinct patterns of WM alterations (Wang et al., 2020). By capturing higher-order relationships among features, CCA extends beyond univariate methods such as TBSS or single-modality approaches, offering deeper insights into the complex interplay of microstructural changes in preterm birth. For further details about the three approaches, their strengths and weaknesses, and their application to this clinical scenario, please refer to Supplementary Section 1.2.

To sum up, this study adopts a multi-faceted approach to understanding the biological phenomenon of prematurity through the following objectives: (i) systemic assessment through diverse dMRI models: leverage multiple HARDI-based models to explore WM microstructure comprehensively, capturing a wide range of microstructural changes beyond traditional metrics; (ii) complementary analytical strategies: combine inference (TBSS), prediction (SVM), and multimodal integration (CCA) to identify significant WM alterations, classify preterm vs. term cohorts, and uncover cross-metric relationships; (iii) bridging inference and prediction: evaluate the alignment and divergence of inferential and predictive approaches in characterizing prematurity-related WM changes, emphasizing their combined value in biomedicine.

By integrating these complementary analytical approaches, the present study emphasizes the importance of utilizing diverse analytical tools to uncover predictive and mechanistic insights. This all-encompassing exploration not only highlights the distinct contributions of inference and prediction but also serves as a model for tackling complex biological phenomena. This comprehensive approach positions the study as a critical step toward developing non-invasive biomarkers and personalized intervention strategies for preterm infants.

## 2 Methods and materials

### 2.1 Subjects

A total of 46 preterms and 23 term-born subjects were enrolled between November 2017 and August 2021 at the Neuroradiology Unit of Gaslini Children's Hospital. Conventional MRI and HARDI were performed using a 3.0 T MRI scanner (Ingenia Cx, Philips, Best, the Netherlands) with a 32-channel head array coil.

To minimize macroscopic movement artifacts, all recommended guidelines for pediatric imaging have been adopted. To protect infants from acoustic disturbances caused by MR sequences, we used baby earmuffs and silicone paste for hearing aids. Furthermore, we avoided most of the motion by swaddling infants and by placing airbags around the babies' head. In addition, protective pads have been placed between the magnet and the patient. All these factors contribute to creating a comfortable and warm rest environment, minimizing the chance of free movement. MRI was performed when possible during spontaneous sleep by administering breast milk or formula about 30 minutes before the start of the exam. In case of spontaneous sleep failure, to minimize macroscopic movement artifacts, the instrumental examination was performed under mild sedation by orally administering midazolam at 0.1-0.2 mg/kg diluted in a 33% glucose solution, subject to signature of informed consent from parents and applied by expertly trained nurses. The exclusion criteria included relevant motion artifacts, oblique positioning, an incomplete imaging process, or a low Signal-To-Noise Ratio (SNR).

Gestational Age (GA) was used as a classifying variable for preterm (GA < 37 weeks) and term birth (GA ≥ 37 weeks). We retrospectively identified all preterm neonates with birth weight <1500 g or at risk (for instance, those with anemia or intrauterine growth restriction) who underwent brain MRI at Term-Equivalent Age (TEA) in the setting of our institutional screening program for identification of prematurity-related lesions. At term, neonates underwent brain MR imaging for clinical indications, including minor trauma, suspect meningitis, and transient neurologic symptoms and signs; all had normal brain anatomy and neurologic examination. Details of the subjects demographics are reported in Table 1.

**Table 1:** Demographic features of neonatal brain.

	**Preterm neonates (*n* = 46)**	**Term-born neonates (*n* = 23)**
Gender (M/F)	14/32	15/8
Mean GA (range; week)	31.15 ± 2.54 (25.29–36.71)	39.13 ± 1.49 (37.00–41.71)
Mean PNA (range; week)	8.05 ± 3.26 (1.43–14.29)	2.50 ± 2.48 (0.14–10.14)
Mean PMA (range; week)	39.21 ± 2.49 (32.86–47.57)	41.63 ± 2.61 (34.00–48.43)
Mean HC (range; cm)	28.72 ± 2.52 (24–34)	34.13 ± 1.55 (29.00–37.00)
Mean BW (range; g)	1,581.98 ± 626.92 (730.00–3,790.00)	3,117.48 ± 510.82 (2,270.00–4,096.00)

M/F, number of male and female infants; GA, gestational age; PNA, postnatal age; PMA, postmenstrual age; HC, head circumference; BW, birth weight.

#### 2.1.1 Ethics approval

This single-center study was carried out in accordance with the recommendations of the Comitato Etico Regione Liguria, Genoa, Italy, with written informed parental consent obtained for each infant prior to examination in accordance with the Declaration of Helsinki.

### 2.2 MR acquisition

Our acquisition protocol included Turbo Field Echo (TFE) 3D T1-weighted and HARDI sequences. Details of the acquisition are reported in Table 2.

**Table 2:** Acquisition protocols for structural T1 and HARDI series.

	**3dT1**	**HARDI**
TR/TE (s)	0.6/0.026337	2.086/0.114
Diffusion scheme (s/mm^2^)	-	5 b = 0, 30 b = 700, 60 b = 2800
Flip angle (°)	90	90
Reconstruction resolution (mm)	0.38*0.38	1.5*1.5
Reconstruction matrix	512*512	144*144
Multi-band factor	-	2
# Averages	2	1
Slice thickness (mm)	0.5 without gap	2.2, without gap
Slice orientation	Sagittal	axial
# Slices	251	42
Total scan time	4 min 5 s	3 min 30 s
Partial Fourier Factor	-	0.6

### 2.3 Preprocessing pipeline

#### 2.3.1 Structural images

**Figure 1:**
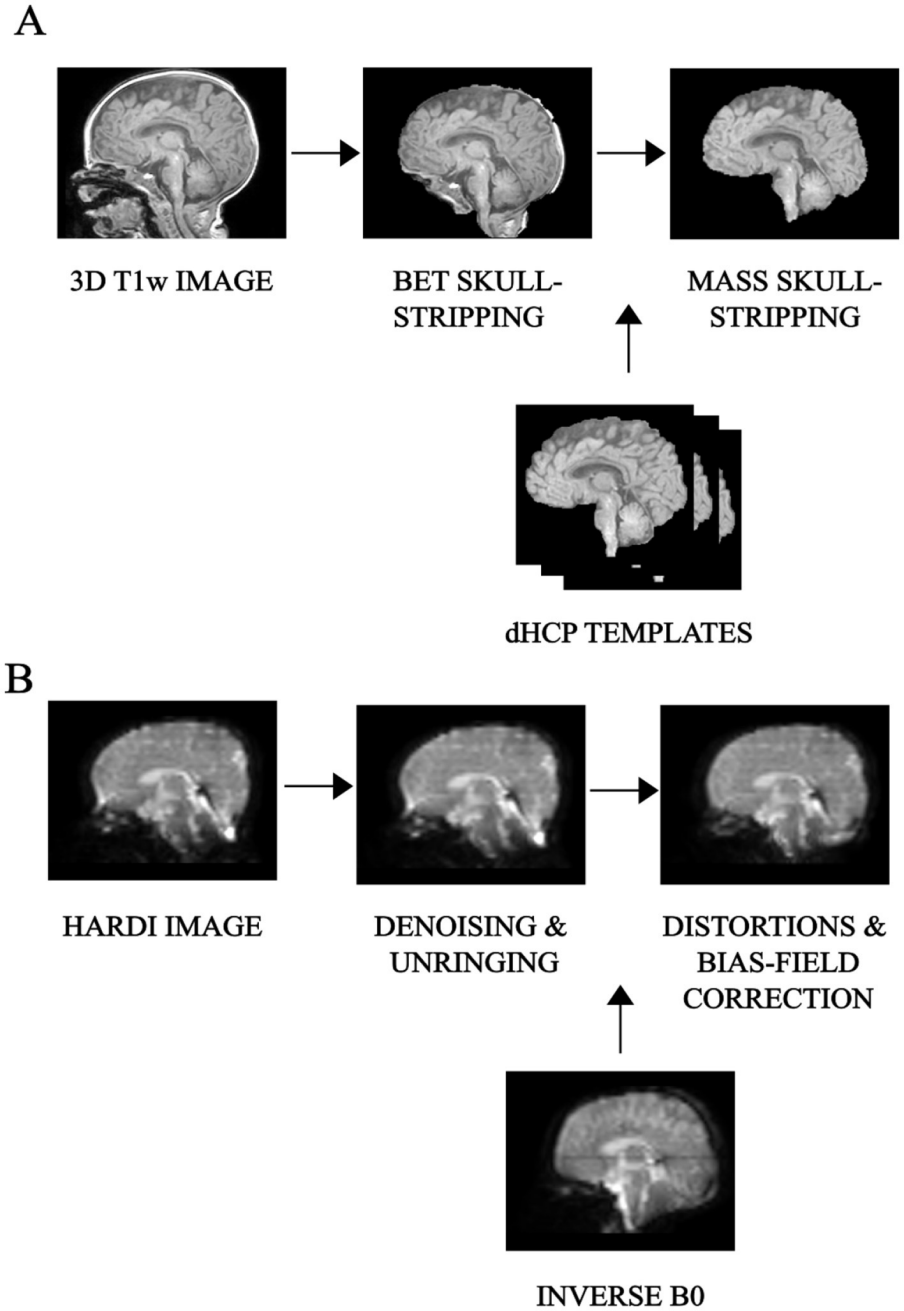
Preprocessing pipeline: overview of the main preliminary image processing steps performed on **(A)** 3D T1-weighted, whose key step is skull-stripping and **(B)** HARDI scans, whose core is represented by denoising as well as distortion correction, for an example subject.

The first critical step was skull-stripping. When dealing with neonatal scans, standard skull-stripping methods (Hosseini et al., 2015; Smith, 2000; Iglesias et al., 2011; Shattuck and Leahy, 2000) failed to correctly remove non-brain areas, thus requiring manual corrections and introducing both a user- and a subject-based bias. Therefore, we opted for Multi Atlas Skull Stripping (MASS) (Doshi et al., 2013), which performs brain extraction through a template selection strategy, obtaining a higher (around 10%) accuracy than recent state-of-the-art tools and avoiding user intervention. As a preliminary step, 3D T1-weighted images were FOV-reduced, processed with Brain Extraction Toolbox (BET) (Smith, 2000), and then bias-field corrected with the N4 algorithm to suppress low-frequency inhomogeneities (Tustison et al., 2010). At this phase, under the supervision of a board-certified neuroradiologist, we selected six subjects that best represented the anatomical variations within the dataset and processed this cohort with the developing Human Connectome Project (dHCP) pipeline (Hughes et al., 2017). The six 3D T1-weighted brain-extracted images generated with the dHCP pipeline were subsequently used as a reference template to train the MASS algorithm. A final re-run of the N4 algorithm ensured bias-field correction using the correct mask extracted with the MASS framework instead of the rough one after preliminary brain extraction with BET. All preprocessing procedures relative to the structural scans are summarized in Figure 1A.

#### 2.3.2 HARDI scans

HARDI scans in pediatrics are sensitive to low SNR and are more prone to macro as well as micro sources of movement. We thus used Patch2Self denoising (Fadnavis et al., 2020) as the very first preprocessing step for diffusion imaging. This denoiser turned out to be particularly suitable for higher-order diffusion models, outperforming other existing methods at visual and modeling tasks (Schilling et al., 2022). The method was implemented using DIPY v.1.4.0 (Garyfallidis et al., 2014) and applied with an OLS regressor, with the threshold for *b* = 0 shell at 100, given the variability of non-diffusion-weighted *b* values. All subsequent preprocessing steps were done in Mrtrix3 v.3.0.1 (Tournier et al., 2019). The standard analysis pipeline performed well also on neonatal scans thanks to overall good image contrast—(i) denoising; (ii) unringing; (iii) Echo Planar Imaging (EPI)—distortion correction (with reversed phase-encoding on b=0 s/mm^2^), eddy-current and movement distortion correction; (iv) B1-field inhomogeneity correction. All preprocessing steps relative to the diffusion images are displayed in Figure 1B.

Regarding co-registration of structural and diffusion scans, for each subject, the mean b=0 image from the diffusion data was registered to the 3D T1-weighted structural image using a rigid-body transformation in FSL (Jenkinson et al., 2012), due to their high degree of overlap. The resulting inverse transformation matrix was exploited to map coordinates or data from the T1 space back to the diffusion space. This allowed subsequent analyses to be carried out in the native diffusion space of each subject, avoiding manipulation or distortion but also maintaining the inherently higher resolution of structural images.

#### 2.3.3 Microstructural models

All quantitative diffusion features in this study result from fitting a different model to the measured dMRI signal on a voxel-wise basis. Despite the multiplicity of existing microstructural dMRI models, the majority fall under the category of linear models fitted with linear least-squares, hence the redundancy of information concealed in diffusion measures. More specifically, all these models share the representation of the dMRI signal as an expansion in an appropriately chosen functional basis, where the coefficients are determined using some variation of least squares (Sjölund et al., 2018). In virtue of this, in the absence of noise, they all can be traced back to the same mathematical equation:


(1) $$\begin{array}{c} y(x)=\overset{d}{\underset{i=1}{\sum }}c_{i}\phi_{i}(x) \end{array}$$

where *y* is the response variable to be modeled, *x* is a single measurement, and ϕ_*i*_(*x*) is the (possibly nonlinear) function with the corresponding coefficients *c*_*i*_. In practice, given *N* observations (*x*_*j*_, *y*_*j*_), we aim to estimate $\hat{c}$ such that $y\approx \Phi \hat{c}$, where we have introduced the matrix Φ_*ji*_ = ϕ_*i*_(*x*_*j*_).

**Figure 2:**
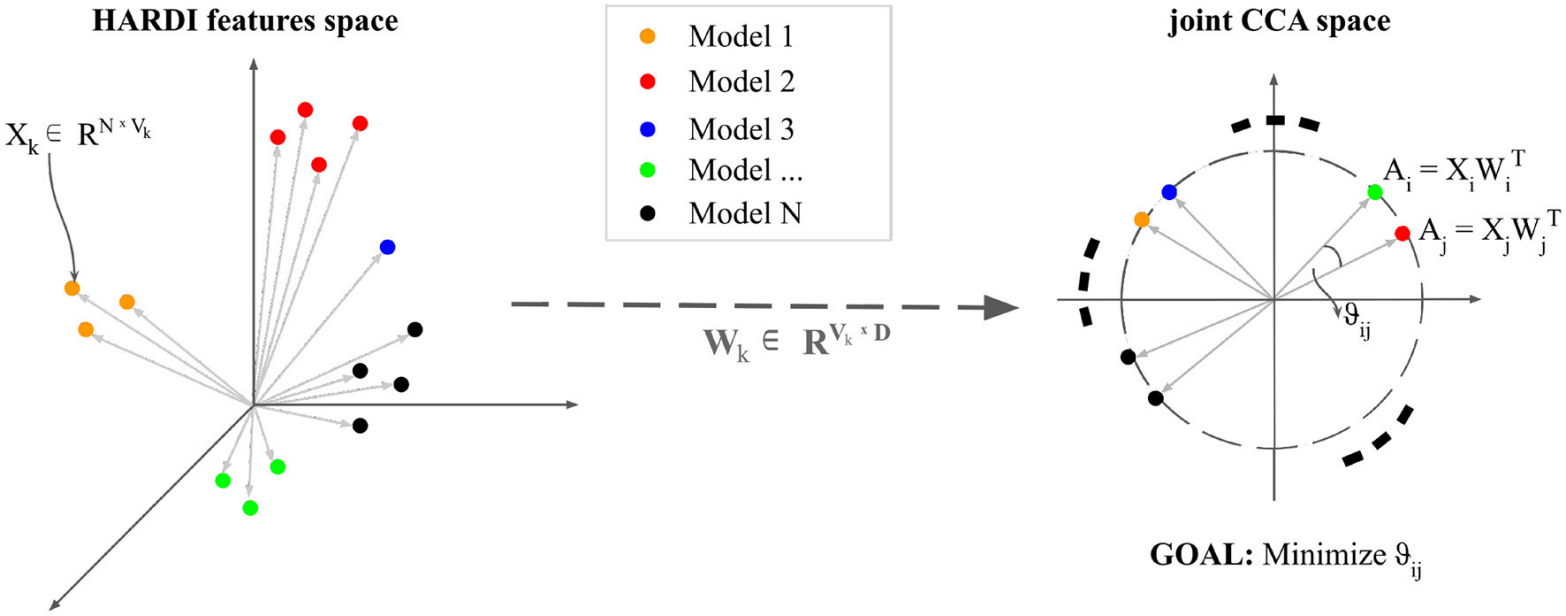
An intuitive visualization of Canonical Correlation Analysis: Let *N* be the number of observations. *n* datasets—variable depending on each diffusion model—X_*k*_ ∈ ${\text{R}}^{N\text{x}V_{k}}$ are transformed by projections W_*k*_ ∈ ${\text{R }}^{V_{k}\text{x}D}$ such that each paired embedding (A_*i*_, A_*j*_) is maximally correlated with unit length in the projected space.

Multivariate CCA analysis has been applied precisely to further investigate how each of these diffusion features relates to each other, given their common starting mathematical formulation (Figure 2).

**Figure 3:**
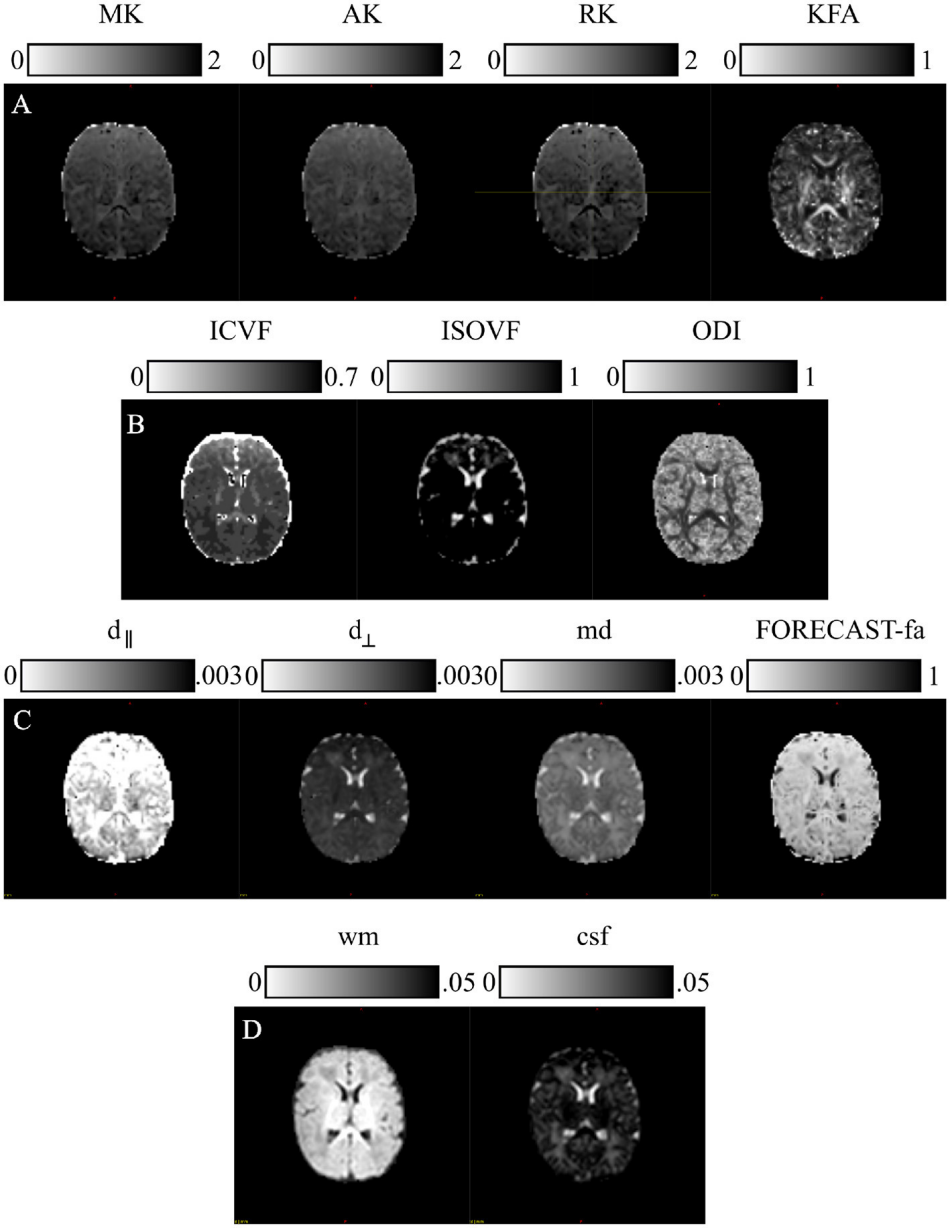
Microstructural models: parametric scalar maps derived from all the HARDI models employed for this study: **(A)** Diffusion Kurtosis Imaging (DKI), **(B)** Neurite Orientation Dispersion and Density Imaging (NODDI), **(C)** Fiber Orientation Estimated using Continuous Axially Symmetric Tensors (FORECAST), **(D)** Multi-Shell Multi-Tissue Constrained Spherical Deconvolution (MSMT CSD).

These microstructural dMRI models have been easily utilized for this cohort thanks to the overall high image quality (Figure 3). The outcome produced by each model has been inspected by two experienced pediatric neuroradiologists (DT and MS) with 10 and 15 years of experience, respectively, and compared with existing studies on age-matched cohorts. Furthermore, to avoid spurious contributions from non-representative image portions and to reduce computational time, all models have been applied to a masked version of the data derived from averaging and skull-stripping the non-diffusion weighted pre-processed volumes. Further details about each specific HARDI microstructural model are provided in Supplementary Section 1.1.

##### 2.3.3.1 Diffusion Kurtosis Imaging

We estimated DKI maps using DIPY v.1.4.0 (https://dipy.org) (Garyfallidis et al., 2014). Standard parametric maps—Mean Kurtosis (MK), Axial Kurtosis (AK), Radial Kurtosis (RK), and Kurtosis Fractional Anisotropy (KFA)—were thus generated. Since these measures are susceptible to high-amplitude outliers, we removed their impact by limiting the extraction of metrics within the typical range (0, 3).

##### 2.3.3.2 Neurite orientation dispersion and density imaging

We computed NODDI-related measures—Intra Cellular Volume Fraction (ICVF), ISOtropic Volume Fraction (ISOVF), and Orientation Dispersion Index (ODI)—with a linear framework for Accelerated Microstructure Imaging via Convex Optimization (AMICO) implemented in Python (https://github.com/daducci/AMICO), which, through a convex optimization approach, drastically accelerates the fit of advanced dMRI techniques while preserving accuracy and precision in the estimated parameters, thus meeting real application demands (Daducci et al., 2015).

##### 2.3.3.3 Fiber Orientation Estimated using Continuous Axially Symmetric Tensors

We resorted to DIPY also for the computation of measures derived from the FORECAST model (Anderson, 2005; Kaden et al., 2016). We used 6 as the spherical harmonics order (*sh*_*order*) for the fiber Orientation Distribution Function (fODF) and CSD as the spherical deconvolution algorithm for the FORECAST basis fitting (*dec*_*alg*) to extract crossing invariant tensor indices. These are mean diffusivity (md), perpendicular diffusivity (d_⊥_), parallel diffusivity (d_∥_), and fractional anisotropy (FORECAST-fa). Using all b-value shells with a basis order of 6 fully leverages the available diffusion-weighted information across varying diffusion sensitivities. This configuration effectively captures both large-scale orientations and fine microstructural details, making it well-suited for robust and computationally efficient studies of complex white matter architecture (Raffelt et al., 2012; Jeurissen et al., 2014; Tournier et al., 2007; Anderson, 2005).

##### 2.3.3.4 Multi-Shell Multi-Tissue Constrained Spherical Deconvolution

Application of MSMT CSD has been performed in MRtrix3 (http://www.mrtrix.org/). For response function estimation, used as the kernel by the deconvolution algorithm, we resorted to the *dhollander* approach, suitable for computing MSMT response functions in the case of multi-tissue variants of SD and more reliable in the case of neonates (Dhollander et al., 2016, 2019). We also maintained the default spherical harmonics order in MRtrix3's MSMT CSD implementation to achieve an optimal balance of angular resolution and noise resilience. This choice aligns with best practices for neonatal HARDI data and leverages MRtrix3's robust, validated parameter defaults to ensure consistency and reliability in diffusion modeling (Tournier et al., 2007; Jeurissen et al., 2014). However, given the poor WM/Gray Matter (GM) contrast inherent to neonatal scans (Dhollander et al., 2018), we were limited to extracting tissue-specific ODF just for WM and Cerebro-Spinal Fluid (CSF). Moreover, since we were interested in performing population studies, we used the same response function for all our cohorts. To this end, we calculated the average tissue response function for all subjects exclusively for WM and CSF responses, named wm and csf, respectively.

#### 2.3.4 Univariate statistics

##### 2.3.4.1 FA skeleton generation

We first used TBSS, a widely used voxel-wise statistical inference for WM anatomy (Bach et al., 2014), to inspect potential per-voxel differences across microstructural-derived markers typical of preterm birth compared to term-born controls. However, once again neonatal imaging caused the standard TBSS pipeline developed in FSL (https://fsl.fmrib.ox.ac.uk/fsl/fslwiki) to present technical challenges due to smaller anatomical dimension and lower image contrast and resolution. We thus integrated it with DTI-TK (http://dti-tk.sourceforge.net/pmwiki/pmwiki.php?n=Documentation.TBSS), as suggested also in Bach et al. (2014) and Tokariev et al. (2020).

The latter is a spatial normalization and atlas construction toolkit optimized for examining WM morphometry through tensor-based registration able to leverage rich discriminating features.

**Figure 4:**
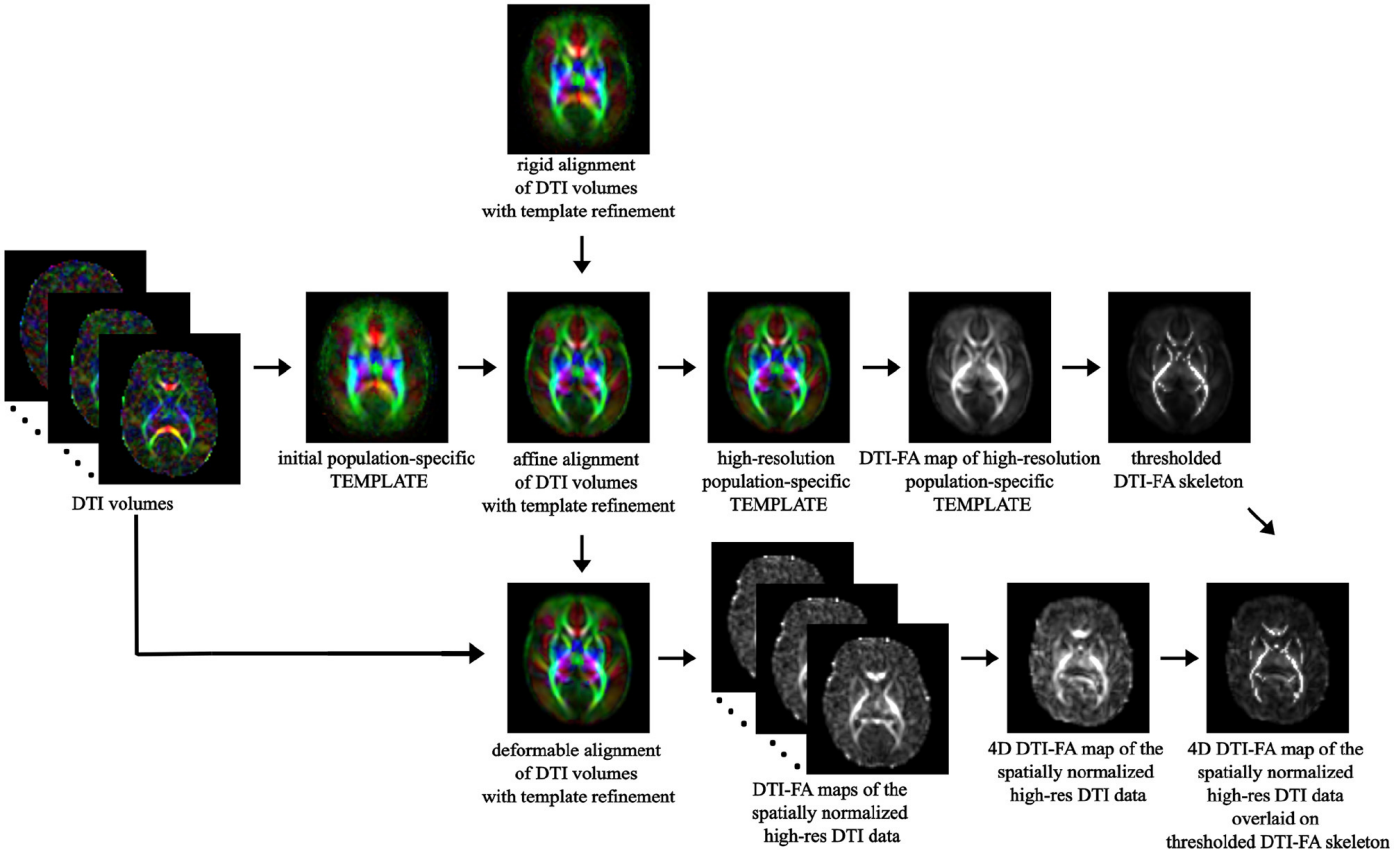
TBSS pipeline: overview of the main steps of the TBSS framework, from spatial normalization of DTI volumes to bootstrapping the within-population template to skeletonization of the template DTI-FA map and projection of each subject's DTI-FA onto the skeleton.

The main differences from the standard TBSS pipeline are: (i) limiting DTI tensor computation through FSL to the *b*=700 s/mm^2^ shell rather than the whole multi-shell diffusion volume; (ii) at the registration phase, bootstrapping a population-specific DTI template from our whole cohort of study without relying on an existing one to better capture within-population features (Supplementary Figure S1); (iii) thresholding the resulting WM skeleton of the high-resolution population-specific DTI template at 0.1 level, in agreement with other works on neonates (Ball et al., 2010) (Figure 4).

##### 2.3.4.2 Non-FA metrics

In order to extend TBSS analysis to diffusion-derived measures other than DTI-FA, we repeated DTI-TK + TBSS steps, similar to what was done in Timmers et al. (2016). Specifically, these non-FA metrics include DKI- (MK, AK, RK, KFA), NODDI- (ICVF, ISOVF, ODI), FORECAST- (md, d_∥_, d_⊥_, FORECAST-fa), and MSMT CSD (wm, csf)—derived measures, respectively. We thus converted each microstructural scalar map to the DTI-TK coordinates, and then we reapplied to each measure the previously obtained nonlinear registration transform to transfer each DTI-FA map to the population-specific template. This procedure was repeated for each of the microstructural measures analyzed in this study.

#### 2.3.5 Univariate predictive model

##### 2.3.5.1 Machine Learning methods for classification

Moving to ML analysis, we performed preterm/term-born subject classification based on a predictive model.

Given the small amount of data available to train our model, we thus resorted to an SVM framework to categorize preterm-born and term-born individuals based on the whole-brain WM skeleton estimated using TBSS. Indeed, among the variety of predictive techniques applied so far in neuroimaging settings, SVM has emerged as one of the most effective methods (Chin et al., 2018; Chu et al., 2015) in coping with high-dimensional data and providing good classification results (Vapnik, 1999).

We also carried out a further analysis to investigate how the performance changes by varying the input dimension of our data through feature selection, and then we trained a classification model based on related findings. For the implementation of ML methods, we resorted to *scikit*-*learn* free software in Python (https://scikit-learn.org/stable/).

##### 2.3.5.2 Experimental design

**Figure 5:**
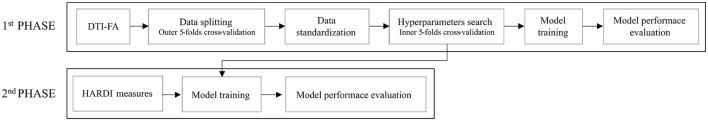
Experimental design for SVM classification: in a first phase, an SVM classification estimator is chosen to best perform on DTI-FA skeletonized data; in a second phase the, selected model is extended to other non-FA measures.

The experiments we carried out can be subdivided into two phases (Figure 5).

In the first phase, we adopted SVM to perform binary classification starting with the DTI-FA map, computed through DIPY v.1.4.0, warped to common TBSS space, and masked by the thresholded WM skeleton for all 69 infants involved. We then split the dataset into learning and testing by stratified 5-fold cross-validation (*outer*-*CV*) to increase the numerosity of our dataset while preserving the same class ratio throughout the *K* folds as the ratio in the original dataset. For each fold, we thus applied data normalization in the default range [0,1] on both the learning set and the test sets. We then further split the learning set into training and validation sets, named *inner*-*CV*, to exhaustively tune the model hyperparameters with the GridSearchCV instance. We thus looked for the best hyperparameter grid by choosing the one that produced the lowest prediction error. This set included: (i) the best penalty term *C* (among 0.001, 0.01, 0.1, 1, 10, 100, and 10^6^); (ii) the best kernel (among linear, radial basis function and polynomial with default degree=3); and (iii) the optimal number of features (selecting 20%, 40%, 60%, 80%, and 100% of the input dataset with the SelectKBest method). For each combination of hyperparameters, we fitted a model on the training set and thus evaluated its performance by computing the average F1 score across folds on the validation set. By selecting the set of parameters whose average F1 score was the best, we then trained such an SVM model on the learning set and subsequently evaluated its performance in terms of average and standard deviation of accuracy, precision, recall, F1 score, and Area Under the Receiver Operating Characteristic (ROC) curve (AUC) across folds on the unseen test set.

In the second phase, once selected the model classifier offering the best performance on DTI-FA data was identified, we further evaluated the classification performance when giving as inputs the parametric measures from other microstructural models than DTI. In this phase, we did not perform any *inner*-*CV* as we did not introduce a hyperparameter search. The decision not to re-optimize the classifier was justified by the desire to maintain a controlled comparison between the series of measurements, using the hyperparameters from the first stage. Indeed, this approach minimizes variability by focusing on how different microstructural measures affect model performance. Conversely, for each input variable, we again carried out the *outer*-*CV* to provide a more robust evaluation of the model. We thus trained the model on the learning set and then assessed the model on the test set, computing the average and standard deviation of usual scores.

#### 2.3.6 Weight maps extraction and comparison with TBSS

Finally, to relate the results from inferential speculation with those from prediction, we extracted weight maps from the selected SVM classifier within *outer*-*CV*, averaged them across the 5 folds, normalized them between 0 and 1, and reshaped them as the 3D input TBSS skeleton for mere visual comparison. The weights are SVM coefficients determining the discriminant hyperplane, which depicts the relevance of each voxel for classification between positive and negative conditions.

We thus computed the standard Pearson's correlation between the normalized SVM weight maps and TBSS normalized significance maps (*p*-maps) for each of the microstructural measures analyzed. To further inspect the overlap between WM discriminating features detected by ML and TBSS, we related Pearson's correlation with the Wasserstein Distance (WD) metric to quantify the distance between the two distributions. Both measures have been computed via the Python library *scipy*.

#### 2.3.7 Multivariate predictive model

Moving to multivariate analysis, the CCA method is based on establishing linear relationships between two or more sets of variables to find out inter-subject co-variances. CCA looks for two or more sets of transformed variates—Canonical Components (CCs) or Variates (CVs)—to assume maximum correlation across the two datasets while being uncorrelated within each dataset. Details about its mathematical formulation are provided in the Supplementary Section 1.2.

In our study, we resorted to the open-source Python package *Pyrcca* (Bilenko and Gallant, 2016) to perform a multi-set CCA based on fusing all advanced dMRI models under analysis (Supplementary Figure S2). We used as input all 14 HARDI measures after filling in missing values and z-scoring. A linear kernel was used to reduce the computational complexity of the analysis. Moreover, we opted for a regularized kernel CCA to avoid overfitting, given the low numerosity of our datasets, and to relax the orthogonality constraint between the CCs. Finally, we estimated the optimal set of CCA hyperparameters—the regularization coefficient and the number of CCs—empirically by using GridSearchCV. Specifically, the optimal regularization parameter was chosen from a logarithmically spaced range of 10 values between 1 × 10^−4^ and 1 × 10^2^, while the optimal number of components was chosen between 1 and 5. We selected these ranges based on pilot analyses performed on an independent dataset that was not used for this publication.

##### 2.3.7.1 Shared/distinct abnormalities

As in Sui et al. (2013), we inspected group differences between the two cohorts by performing a non-parametric Mann-Whitney U Test between each pair of CCs to look for the variates showing abnormalities associated with preterm birth. The statistical survey was followed by the Benjamini-Hochberg correction method for multiple comparisons (Benjamini and Hochberg, 1995). If the components show group differences in more than one dMRI model, they are called modality-common or joint group-discriminative CVs. Conversely, if the components show group differences only in a single model, they are called modality-unique group-discriminative CVs.

##### 2.3.7.2 Inter-modality correlation

We then investigated the inter-correlation existing between microstructural dMRI models by looking at the Canonical Correlation Coefficients (CCC) to establish whether the joint-group discriminative components additionally have strong inter-modality correlation, which would reflect the interaction and correspondence among diffusion imaging techniques.

## 3 Results

### 3.1 TBSS analysis exhibits a significant decrease in preterm subjects for a subgroup of HARDI measures

**Figure 6:**
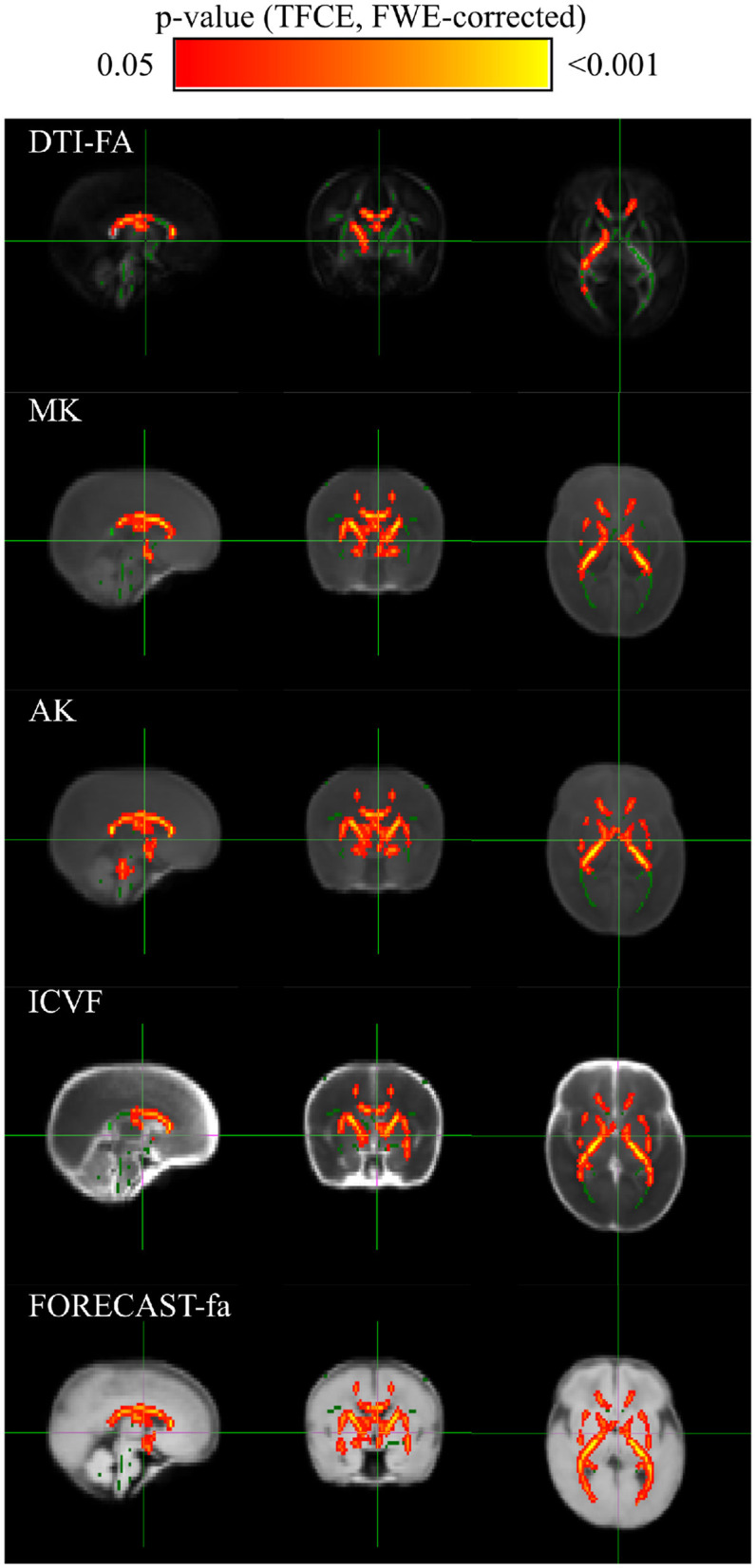
TBSS exhibits discriminant white matter areas for a subset of microstructural measures: group-level voxel-wise statistical difference maps for DTI-FA (FA), Mean Kurtosis (MK), Axial Kurtosis (AK), IntraCellular Volume Fraction (ICVF) and FORECAST fractional anisotropy (FORECAST-fa) between preterm and term-born cohorts. Green indicates the DTI-FA skeleton with a threshold of 0.1, which highlights the tracts used in the comparison. Red-Yellow indicates the regions with decreased metrics values in the preterm group after an unpaired voxel-wise *t*-test with Family-Wise Error (FWE)-corrected *p*-values using Threshold-Free Cluster Enhancement (TFCE).

Cross-subject voxel-wise TBSS statistics unraveled significantly different voxels exclusively on a subset of the microstructural maps under consideration, using an unpaired voxel-wise t-test with Family-Wise Error (FWE) correction using Threshold-Free Cluster Enhancement (TCFE) (Smith and Nichols, 2009). Specifically, compared with the term cohort, the preterm group showed a significant decrease in DTI-FA, MK, AK, ICVF, and FORECAST-fa. The WM regions with significant between-group differences in diffusion metrics are shown in Figure 6. Conversely, no significant differences were observed by TBSS analysis in RK, KFA, ISOVF, OD, MD, d_∥_, or, d_⊥_, or in MSMT-derived measures. Table 3 summarizes the significant clusters identified by TFCE in WM regions where diffusion metrics showed decreased values in the preterm group compared with the term group, highlighting metric-specific spatial patterns and degrees of sensitivity to WM microstructural alterations associated with prematurity.

**Table 3:** Summary of significant clusters from TFCE in white matter regions showing reduced values in diffusion metrics in the preterm group compared with the term group.

**Metric**	**Cluster size (voxels)**	**Peak value**	**Max location (MNI)**	**Corrected *p*-value**
**DTI-FA**				
Genu, body, and splenium of CC	182	0.978	(102, 94.5, 83.2)	*p* < 0.022
Right IC, CR	79	0.968	(104, 110, 65.2)	*p* < 0.032
Posterior TR	61	0.966	(87, 133, 67.5)	*p* < 0.034
Right EC	8	0.950	(117, 84, 67.5)	*p* < 0.05
**MK**				
Genu, body, and splenium of CC	308	0.968	(117, 98, 69.8)	*p* < 0.032
Right IC, CR, right EC	279	0.968	(78, 98, 65.2)	*p* < 0.032
Posterior TR	226	0.974	(94.5, 126, 81)	*p* < 0.026
**AK**				
CC, bilateral IC and EC	1,118	0.978	(104, 103, 56.2)	*p* < 0.022
Right CR	39	0.950	(79.5, 116, 65.2)	*p* < 0.05
**ICVF**				
Genu, body, and splenium of CC, right IC	409	0.984	(85.5, 105, 67.5)	*p* < 0.016
Bilateral CR, Posterior TR	360	0.990	(108, 94.5, 99)	*p* < 0.01
**FORECAST-fa**				
CC, bilateral IC and EC, anterior CR, posterior TR	1,494	0.998	(76.5, 107, 63)	*p* < 0.002

Regions are identified using white matter tracts and anatomical labels. *Cluster Size* describes the spatial extent of the significant region, measured by the number of significant voxels in the cluster. *Peak Value* is the highest t-statistic value observed within the cluster, indicating the strongest statistical difference in the region. *Max Location (MNI)* specifies the exact coordinates (in MNI space) of the voxel with the peak t-statistic, allowing anatomical localization; *corrected p-value* reflects the significance of the cluster after correcting for multiple comparisons.

CC, Corpus callosum; IC, Internal capsule; CR, Corona radiata; EC, External capsule; TR, Thalamic radiation.

More in detail, compared with the term group, the preterm cohort had significantly decreased DTI-FA values in widespread WM areas, predominately in the genu, body, and splenium of the corpus callosum; right internal and external capsule, corona radiata, and posterior thalamic radiation. The distribution of areas with decreased MK was similar with respect to the areas with decreased DTI-FA. AK exhibited a pattern analogous to MK whilst comprising a bilateral external capsule. The same applies to the ICVF metric. The amount of WM areas showing a significant decrease in prematurity increased for the FORECAST-fa parameter, which extended to the whole corpus callosum, bilateral internal capsule, external capsule, anterior corona radiata, and, finally, posterior thalamic radiation (including the optic radiation).

### 3.2 SVM classification of group membership achieves good performance, especially in terms of area under the curve score

Since the performance of a model significantly depends on the value of its hyperparameters, we first focused on hyperparameter tuning to determine the optimal values for our classification estimator.

**Figure 7:**
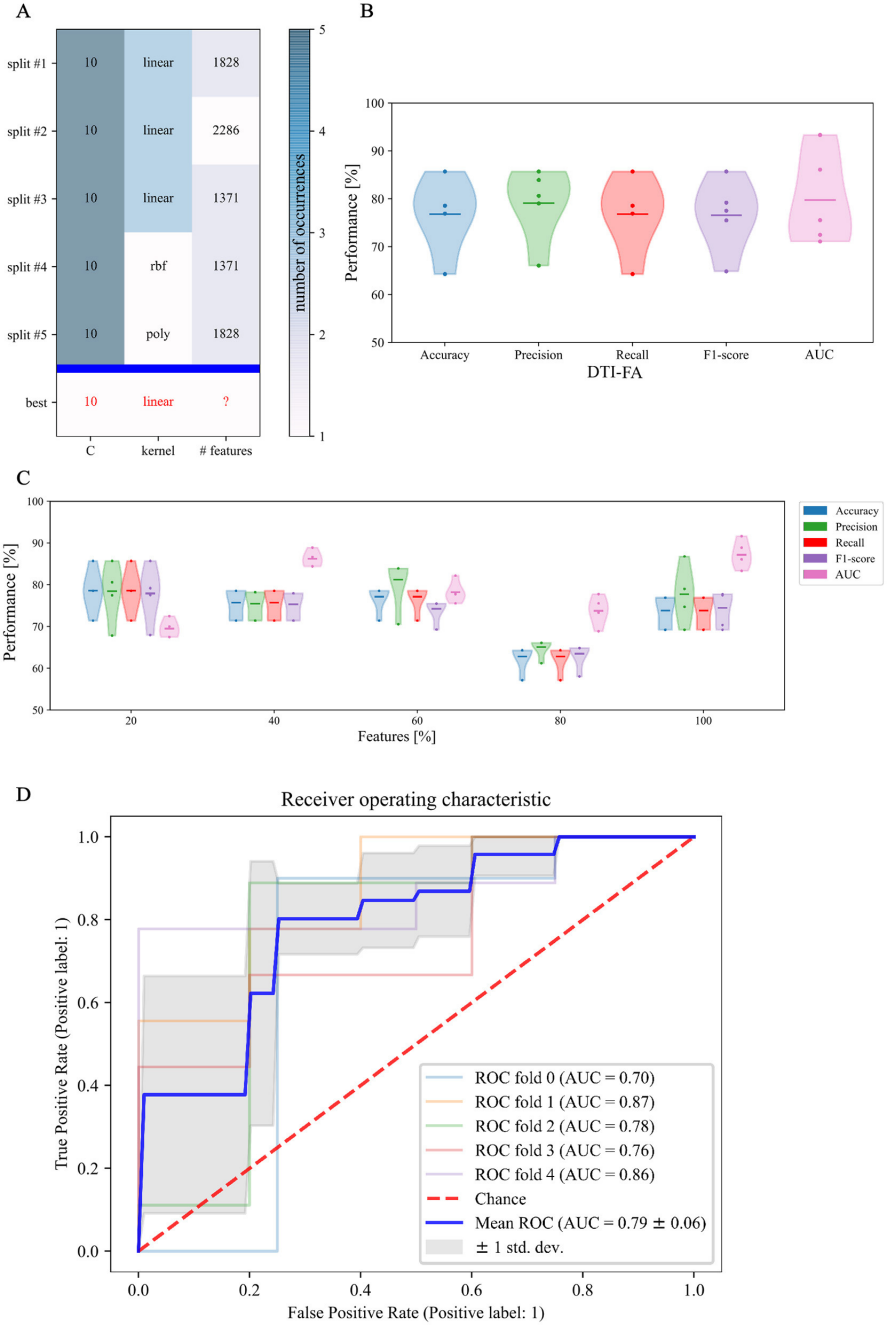
First phase of binary classification: SVM tuning of hyperparameters training on FA skeletonized data: **(A)** cross-validated search of the best set of hyperparameters for our SVM estimator on stratified 5-fold data; **(B)** relative performance for every score across folds; **(C)** Different sets of selected features along with relative performance for every score across folds; and **(D)** area under the ROC curve score.

In this respect, Figure 7A shows the result of the cross-validated grid search over the parameter grid across each of the five folds. Furthermore, based on the selected hyperparameters, we fitted our model on the training set and evaluated its performance on the test set in terms of F1 score, accuracy, precision, recall, and AUC across each of the five-folds (Figure 7B). To establish the best estimator possible based on the input data, we counted how many folds in which a variable was selected and could thus conclude that penalty term *C* and *linear* kernel were the most frequently selected hyperparameters. Conversely, the search turned out to be less stable in terms of the optimal number of features, which varied at every fold (Figure 7C). Therefore, to set the last missing parameter for our estimator, we set *C* and kernel according to their most chosen values while varying the number of features as a percentage of the total amount.

**Figure 8:**
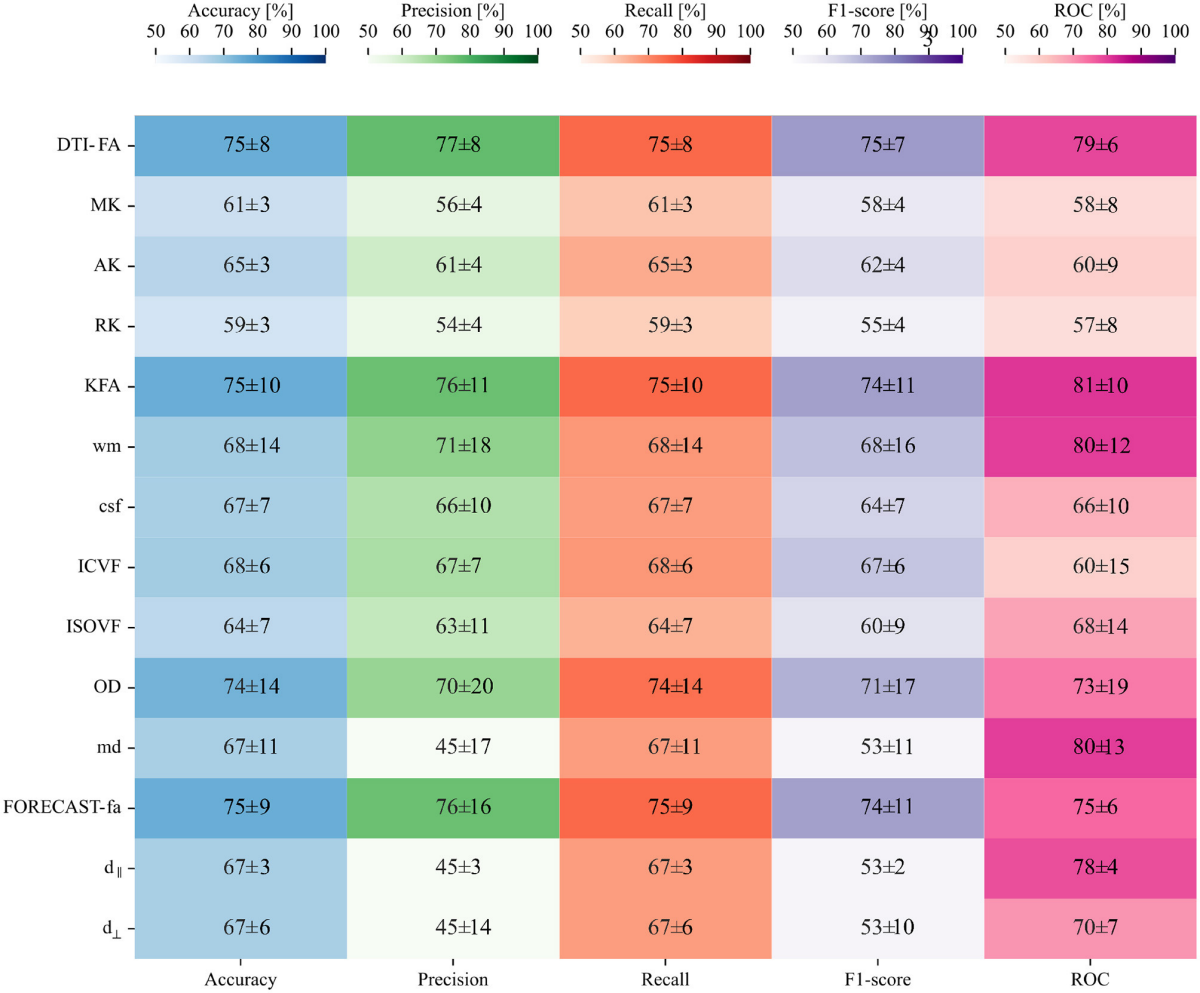
Second phase of binary classification: SVM testing on non-FA skeletonized data on average shows good performance, especially for the AUC score: a heatmap containing the average and relative standard deviation, in percentage, of each score and for all the HARDI measures under analysis.

Figure 7C confirms that, in our case, feature selection is not beneficial for improving classification performance. Indeed, both average value and standard deviation across folds of each score remain constant with variable subsets of features. In addition, the average AUC score proves to be maximal (0.87) when including the whole feature amount. We thus opted for avoiding feature reduction and kept the whole of the features to define the final version of our SVM estimator. As regards this definitive version of the classifier, a detailed plot of the ROC curve profile for every fold is displayed in Figure 7D. We subsequently trained a classification model without hyperparameter search (*inner*-*CV*) using as input variables the metrics derived from other microstructural HARDI models. Performance in terms of F1 score, accuracy, precision, recall, and AUC for the whole set of microstructural parameters, including DTI-FA, is reported in Figure 8. Of note, among the whole set of measures, the ones exhibiting the highest discriminative power in terms of SVM classification are those probing overall anisotropy and directionality of fibers, namely DTI-FA, KFA, OD, and FORECAST-fa, for which all scores overcome 75%, 74%, 70%, and 74% levels on average, respectively.

### 3.3 Comparison between SVM and TBSS reveals a measure-dependent rate of agreement between the two approaches

**Figure 9:**
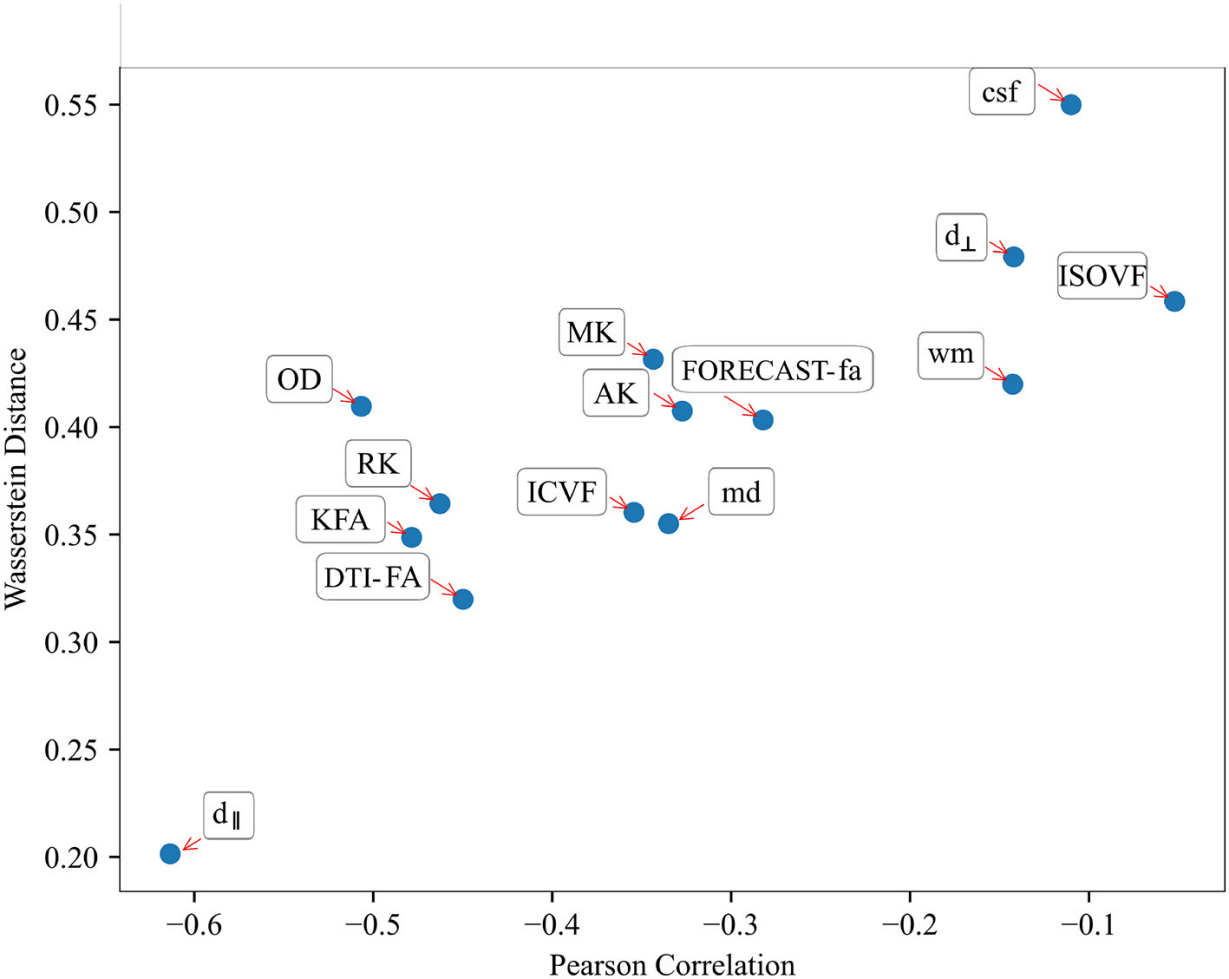
Relationship between Pearson's correlation and Wasserstein Distance shows a good trend of association throughout all HARDI parameters considered: those measures exhibiting the highest absolute correlation values correspondingly have a lower Wasserstein Distance.

Relating variables identified as statistically significant with those identified as predictively relevant, a statistically significant Pearson's correlation for all microstructural measures considered (*p* < 10^−2^) (see Table 4) arose. This relationship was further confirmed by inspecting the association between the absolute Pearson's correlation coefficient and WD, reported in Figure 9, showing a trend of indirect proportionality. Overall, an inverse trend was observed, with measures showing relatively higher absolute correlations generally corresponding to lower WD, although this relationship may vary slightly across specific measures. The correlation was moderate (*r* = 0.61) for the d_∥_ parameter, weakly moderate (*r* ∈ 0.45 − 0.51) for RK, KFA, DTI-FA, and OD, low (*r* ∈ 0.28 − 0.35) for MK, AK, MD, FORECAST-fa, and ICVF, and very low (*r* ∈ 0.05 − 0.14) for d_⊥_, CSD-related measures, and ISOVF (Schober et al., 2018). These results suggest an overall good, though measure-dependent, rate of agreement between *p*-maps derived by the TBSS approach and weights probing the discriminative power of SVM.

**Table 4:** Comparison between inferential TBSS statistics and SVM prediction.

	**Pearson's *r***	***p*-value**	**Wasserstein distance**
DTI-FA	−0.45	<0.0001	0.32
MK	−0.34	<0.0001	0.43
AK	−0.33	<0.0001	0.41
RK	−0.46	<0.0001	0.36
KFA	−0.48	<0.0001	0.35
d_∥_	−0.61	<0.0001	0.20
d_⊥_	−0.14	<0.0001	0.48
md	−0.33	<0.0001	0.36
FORECAST−fa	−0.28	<0.0001	0.40
wm	−0.14	<0.0001	0.42
csf	−0.11	<0.0001	0.55
ICVF	−0.35	<0.0001	0.36
ODI	−0.51	<0.0001	0.41
ISOVF	−0.05	0.013	0.46

### 3.4 Canonical Correlation Analysis unravels joint group differences for parallel diffusivity and ISOtropic Volume Fraction

**Figure 10:**
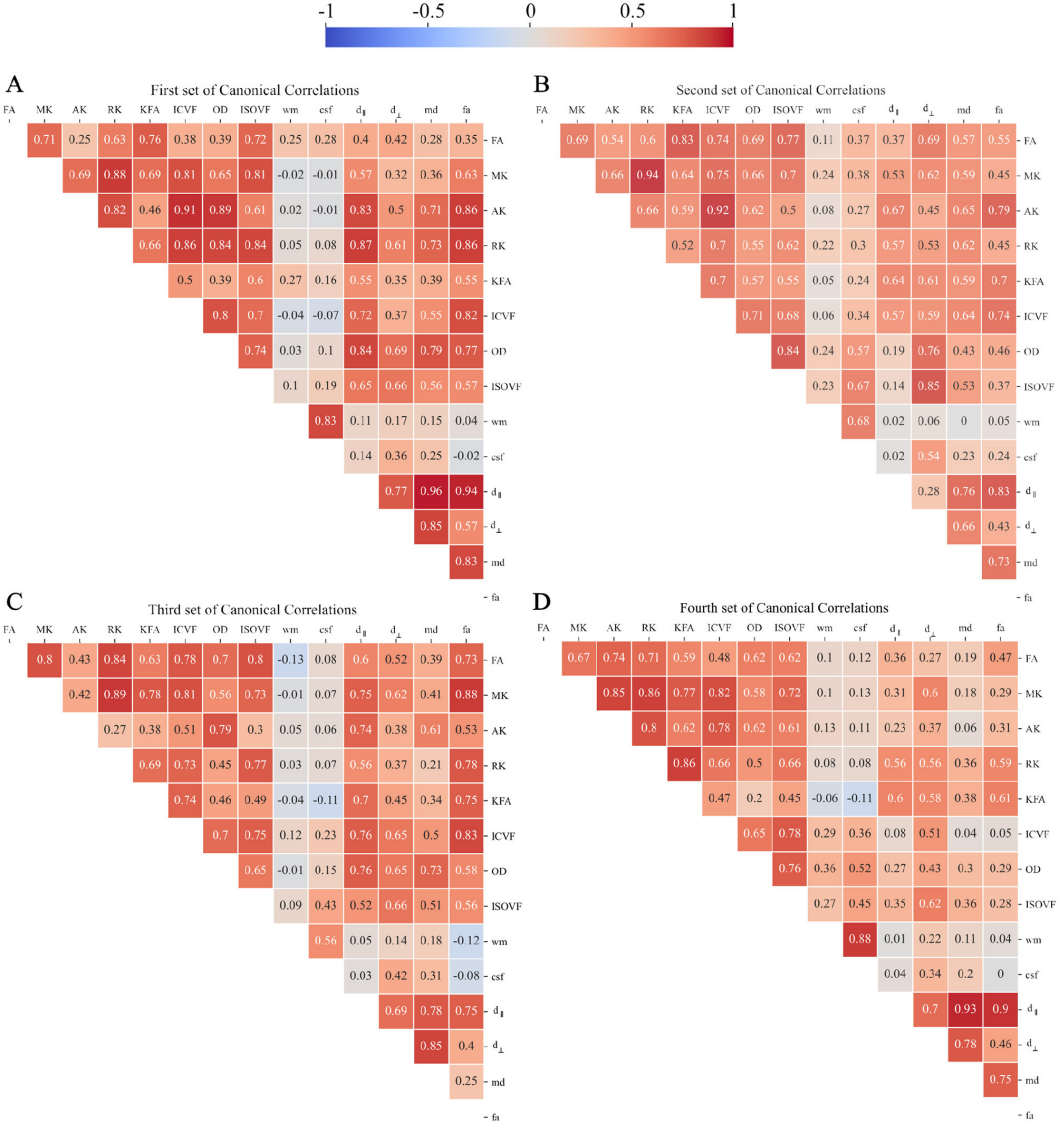
Canonical Correlation Analysis identifies four Canonical Components maximizing pair-wise Canonical Correlation matrices: **(A)** first Canonical Component, **(B)** second Canonical Component, **(C)** third Canonical Component, and **(D)** fourth Canonical Component. For the sake of brevity, *FA* stands for DTI-FA, while *fa* stands for FORECAST-fa.

**Figure 11:**
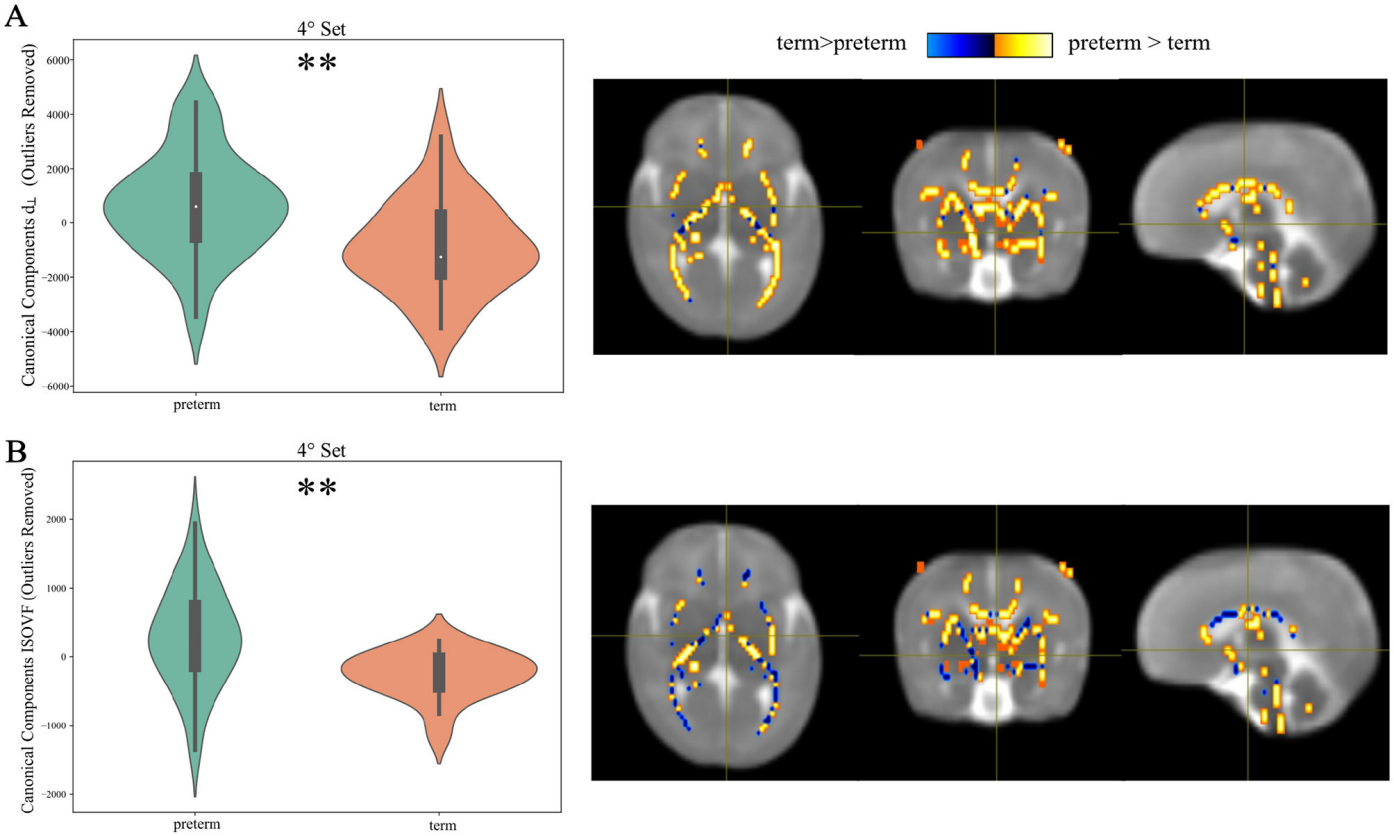
Perpendicular diffusivity (d_⊥_) and ISOtropic Volume Fraction (ISOVF) turn out to be joint group-discriminative Components from Canonical Correlation Analysis: **(A)** Violin plots of the loading parameters for the 4th component for each dMRI measure after outlier removal, with ** indicating the significant *p* values of the Mann–Whitney U Test between term and preterm participants; **(B)** Group-discriminating regions across all modalities. The Z-scored spatial maps exhibit positive Z-values (orange regions), meaning preterm > term subjects, and negative Z-values (blue regions), meaning term > preterm.

Moving to CCA analysis, *Pyrcca* cross-validated hyperparameters search detected the optimal regularization coefficient equal to 0.01, and the optimal number of CVs to 4. Preliminarily, the results of CCA analysis were evaluated in terms of Canonical Correlations to determine the number of meaningful CCs recovered by Pyrcca. Figure 10 contains a heatmap of pairwise correlations between the 14 HARDI measures for each of the 4 sets of CCs. From the Mann–Whitney U Test, CCA analysis applied to our cohort unraveled group differences in the 4th Canonical Component, for which statistically significant differences between preterm and term subjects have been found in ISOVF and d_⊥_ even after outlier removal with the interquartile range method and FDR correction (*p* = 0.014, *U* = 621 and *p* = 0.014, *U* = 759, respectively, α = 0.05), thus being a joint group discriminative independent component. This is depicted in Figure 11A, with violin plots of CVs having statistically significant differences between preterm and term-born subjects. Interestingly, the intramodal connection within the joint-discriminative independent component (4th) indicates a good correlation (*r* = 0.62) (see Figure 10). Furthermore, to visually mark out detected differences between the two groups, we displayed derived spatial maps only for the specific joint group-differentiative CC. In Figure 11B, each z-score-transformed input measure is reported to highlight statistically significant group-discriminating subsets of voxels.

## 4 Discussion

In this work, we examined the complexities associated with preterm birth through a multiplicity of advanced HARDI models in turn employed at different levels of analysis. To the best of our knowledge, this study is thus the first to jointly employ univariate statistics (TBSS) and predictive modeling (SVM) on intramodal advanced dMRI data to comprehensively investigate WM alterations associated with preterm birth. Unlike prior works that have predominantly used these methods independently, our approach leverages their complementary strengths—TBSS for robust group-level analysis and SVM for individualized prediction—providing a multifaceted perspective on WM microstructure. Additionally, for the first time, we integrate CCA to uncover hidden relationships among multiple advanced dMRI models, surpassing traditional statistical tools in capturing complex interdependencies. By combining these methodologies, our study represents a significant advancement toward identifying biologically interpretable and clinically relevant markers of WM alterations in preterm infants, paving the way for more personalized and data-driven approaches in neonatal neuroimaging.

The first tool we considered to investigate the potential characteristics of preterm subjects was TBSS. Through this inferential data analysis strategy, we demonstrated that both DTI-FA and non-FA values can be useful measures to distinguish relevant WM tracts in preterm-born neonates at TEA from term-born controls. It was particularly notable that there was a correspondence between the distribution of areas with decreased DTI-FA and non-FA measures, with an expansion of WM-discriminating areas over the main tracts, especially in the case of beyond-DTI measures. This agrees with existing findings in the literature claiming that: (i) WM maturation is associated with increasing axonal organization, pre-myelination, and myelination, which progressively restricts water diffusion perpendicular to the direction of the axonal fiber; (ii) since premature birth may lead to relatively slow brain development in premature infants, some brain regions are less developed than the full-term infants. This includes the corpus callosum, anterior and posterior limb of the internal capsule, and, more generally, all tracts subject to early myelination whose metabolism is thus vigorous and the oxygen demand is high, which makes these metabolically active areas the first to be damaged in case of risk factors for preterm birth (Ling et al., 2013). For the DTI measures, lower FA has been found across the WM in preterm infants compared with term-born infants (Pecheva et al., 2018; Hüppi et al., 1998; Anjari et al., 2007; Thompson et al., 2011), which correlated with increased prematurity (Ball et al., 2010; Partridge et al., 2004; Ouyang et al., 2019a). Furthermore, WM diffusion measures in preterm infants at TEA have been related to subsequent neurodevelopmental performance. Decreased DTI-FA measures, with an expansion of together with increased MD and RD-FA measures, with an expansion of in the WM at TEA are associated with worsened motor, cognitive, and language performance in early childhood (Counsell et al., 2008; Barnett et al., 2018) as well as visual function (Bassi et al., 2008; Groppo et al., 2014). In Zhao et al. (2021), kurtosis-related parameters, especially MK, were shown to sensitively reflect the brain maturity of premature infants. Decreased MK values were registered in the preterm cohort due to the decreased density of cells and axon membranes associated with impaired brain development.

Similarly, the NODDI model has been applied to investigate WM and GM maturation in the preterm brain (Kimpton et al., 2021; Batalle et al., 2019, 2017; Eaton-Rosen et al., 2015), finding that ICVF increases in the WM with increasing maturation, mainly attributed to increasing axonal growth/density/packing/diameter or pre-myelination/myelination changes, rather than changes in axon coherence or geometry. Moreover, greater ICVF in childhood has been associated with better neurodevelopmental outcomes, IQ (Young et al., 2019; Kelly et al., 2016), visual motor integration (Young et al., 2019), motor, behavioral, and emotional scores (Kelly et al., 2016), language (Mürner-Lavanchy et al., 2018), and maths (Collins et al., 2019). Finally, although not previously investigated in the case of preterm subjects, the FORECAST-fa parameter falls into those measures exhibiting significant differences from preterm to term-born infants, presumably for being the equivalent of the DTI-FA yet far more sensitive to the underlying fiber microanatomy.

The second perspective from which we examined our cohort was an SVM-based approach aimed at a more individualized classification method to overcome shortcomings of group-wise investigations. The good achievement of the SVM in correctly assigning group membership, based on a single MR image, indicates that the distinct brain development of preterm-born individuals can be successfully identified by predictive methods. Indeed, considering the low sample size at disposal, much inferior to the number of features (i.e., image voxels), the SVM classifier managed to handle the issue of overfitting and proved a good performance on both the DTI-FA skeletonized image, on which its model was designed, and on the vast majority of non-FA measures. Specifically, together with DTI-FA, other scalar parameters derived from DKI, NODDI, and FORECAST exhibited good scores in terms of both F1/accuracy and, most importantly, of AUC—a significantly more meaningful measure of classifier performance than accuracy because it does not bias on size of test or evaluation data. Of note, preterm vs. term classification accuracy achieved by the predictive model, however good, was not optimal. This may be due to the diffuse effect of preterm birth on WM microstructure, being optimally captured by methods not requiring anatomically constrained ROIs (Baykara et al., 2016; Blesa et al., 2020). Along with overall good performance scores, the selected classifier also showed strong robustness (i.e., limited variability across folds), another important indicator for model evaluation, assessing its stability. Furthermore, the evidence that the most discriminating features in terms of SVM classification are related to fiber anisotropy stands for dysmaturation or delay in myelination of WM tracts following preterm birth in contrast to term-born controls.

We then explored the relationship occurring between TBSS- and SVM-based methods, assessing the degree of overlap between the two survey methods in attributing relevance to the input variables. The observed negative Pearson's correlation is explained by considering that we compared a significance map from voxel-wise statistics made up of thresholded *p*-values and the map of SVM weight vectors serving as a ranking metric for measuring feature importance (Gaonkar and Davatzikos, 2013). As a result, voxels exhibiting a lower *p*-value correspondingly have a high ranking in the SVM model, which results in the observed inverse trend.

The partial agreement between voxel features identified by TBSS and SVM reflects the complementary strengths of inferential and predictive methods. TBSS excels at identifying group-level differences, while SVM highlights individualized discriminative features, providing a multifaceted understanding of WM alterations.

Such findings are in line with Bzdok et al. (2020), who directly compared explanatory and predictive modeling in both simulated and common real-world datasets, finding out a certain variability in feature identification between the two approaches, with increasing divergence in typical clinical settings. This discrepancy is attributable to the specific data scenario at hand, including properties of the data-generating mechanisms (e.g., available sample size, number of informative input variables, redundancy of information carried in the input variable about the outcome, random noise variation, pathological settings), which affect variable identification in TBSS and SVM in distinct ways. Specifically, small-to-moderate sample size and collinearity between input measures, very common in biological data such as in our case, together with the number of truly relevant variables, commonly unknown in biomedical data analysis in practice, are those driving experimental factors causing the largest disagreements in variable identification.

It is on the premise that integrating multiple datasets from the same participants can increase confidence when making conclusions to a greater degree than traditional statistical approaches (Sui et al., 2013) that we extended our investigation to considering simultaneously multiple microstructural models through CCA. In this study, we investigated brain co-alterations from several advanced dMRI across preterm and term-born cohorts. To our knowledge, this is the first study to clarify preterm birth-related brain changes in different dMRI modalities via an intramodal data fusion model. Specifically, two further measures, d_⊥_ and ISOVF, emerged as relevant markers discriminative of preterm birth, other than those highlighted by TBSS or classification. This proves the capability of CCA to detect potentially hidden relationships between different imaging modalities beyond traditional methods, which could not be detected from a single dMRI model. The brain regions exhibiting the strongest contributions to coherent changes related to preterm birth involve the majority of WM tracts detected with TBSS. More in detail, a simultaneous decrease in both d_⊥_ and ISOVF is observed in the term-born group compared to the preterm one. Such findings are in line with previous studies (Vaher et al., 2022; Barnett et al., 2018; Pecheva et al., 2017; Thompson et al., 2019) and consistent with the higher content of extracellular free water expected in the case of diffuse loss of WM microstructural integrity and organization inherent to preterm birth. Similarly, being d_⊥_ a more sensitive variant of DTI's RD, it proved in turn to be highly reflective of the lack of tortuosity imposed on water motion due to the delayed development of the myelin sheath (Knight et al., 2018).

Through this investigation, we highlight how “importance”, intended as variable relevance, does not have an unambiguous definition across different data analysis strategies. Resorting to *p* values or prediction accuracies for arguing research claims both have flaws and each is insufficient per se. Our findings thus push toward the adoption of a combined approach aimed at exploring similarities and differences of significance and predictability to fully exploit the advantages of both methods in the perspective of a patient-tailored predictive approach, where the goal of forecasting patient-specific disease symptoms in turn helps and complements explaining disease-causing biological mechanisms, finding a common ground between these two apparently opposed methods. In this sense, inference and prediction can be seen as two sides of the same coin, both aimed at understanding and using data to make informed and better decisions.

We are aware that both voxel-wise statistical methods and, in particular, the ML approach benefit from large quantities of data. Our survey is inherently limited by the challenge of collecting clinical data from the targeted population and thus provides preliminary, exploratory insights into microstructural changes associated with prematurity. However, all methodological strategies to handle this issue have been adopted to balance interpretability with predictive robustness, such as the choice of SVM as a classifier and the implementation of nested cross-validation.

Future studies will focus on extending the current dataset and introducing stratification by diagnosis, which could enhance the predictive classification's ability to detect specific WM tract patterns across healthy and pathological preterm subjects. This aligns with our broader goal of developing clinically actionable tools for understanding and monitoring prematurity-related WM changes.

## 5 Conclusion

Results gathered so far from this study revealed that an intramodal dMRI approach can be a valuable tool to distinguish atypical brain microstructure at TEA when compared with a full-term group, regardless of the specific diagnosis based on radiological findings. This differentiation is achieved at three different levels of investigation, to provide a more comprehensive, detailed, and biologically meaningful interpretation of WM microstructure changes associated with prematurity. First, a classical group-level survey tool such as TBSS confirmed the high sensitivity of advanced dMRI methods. Second, a state-of-the-art approach based on SVM classification achieved a high recognition rate. Furthermore, comparing the two methods revealed a distinct agreement in selecting the most discriminating WM regions, mainly depending on the microstructural measure under consideration. Finally, CCA further represents a powerful tool for identifying the inter-measure similarities between metrics associated with preterm birth in a data-driven way, without imposing an explicit model.

Taken together, these insights suggest that combining synergy between modalities and analytical tools will allow for a more thorough investigation of the preterm birth phenomenon providing an unprecedented supplement to our understanding of biological mechanisms. Furthermore, these findings should be added to the body of literature suggesting that there is generalized dysmaturation of the WM in preterm neonates.

Further studies should focus on investigating how well these results generalize to data across centers and on what kind of improvements are needed to reach the end goal of predicting, on an individual basis, the specific outcome of subjects born preterm.

## Data Availability

The raw data supporting the conclusions of this article will be made available by the authors, without undue reservation.
