# Supplementary material for: Multi-view fusion of diffusion MRI microstructural models: a preterm birth study
Source: Front Neurosci. 2024 Dec 20;18:1480735. doi: 10.3389/fnins.2024.1480735 (PMC11695353; doi:10.3389/fnins.2024.1480735)
Supplement: Supplementary file 1 [file Data_Sheet_1.pdf]

Supplementary Material

1 SUPPLEMENTARY DATA

1.1 HARDI Microstrucural Models

1.1.1 Diffusion Kurtosis Imaging

In an attempt to overcome the limitations of the DTI model, several microstructural models have been recently conceived providing a direct link between diffusion properties and specific microstructural features (Assaf and Basser, 2005; Jespersen et al., 2007; Nilsson et al., 2012; Zhang et al., 2012). However, the validity of these models may be compromised by improper assumptions in their complex mathematical formulation (Lampinen et al., 2017; Henriques et al., 2019). An alternative to avoid misleading interpretations is represented by phenomenological models, offering a complete characterization of water diffusion in biological tissues through a simpler mathematical expression containing parameters that do not directly relate to the biological tissue microstructure (Novikov et al., 2018).
Among them, one of the most popular is DKI, an extension of DTI that directly estimates the degree to which water diffusion deviates from a single Gaussian component (Jensen et al., 2005). Indeed, although the DTI model hypothesizes an ideal Gaussian profile for the diffusion of water molecules within every voxel, the presence of physiological boundaries (e.g., cell membranes or myelin sheaths) and obstacles (e.g., organelles, macromolecules) makes this diffusion far from Gaussian. DKI precisely quantifies this excess-kurtosis and is thus able to probe tissue microstructure more faithfully and sensitively (Jensen et al., 2005; Jensen and Helpern, 2010; Fieremans et al., 2011). This, in turn,implies that the scalar measures provided by DKI closely relate to microstructural alterations in both healthy and pathological tissues providing more sensitive and specific markers for tissue injury than DTI data alone (Grossman et al., 2012; Hui et al., 2012; Fieremans et al., 2013; Rudrapatna et al., 2014; Steven et al., 2014; Marrale et al., 2016; Lin et al., 2018; Huber et al., 2019; Zhu et al., 2021). Furthermore, DKI provides more accurate information than DTI for tractography applications by resolving more complex fiber configurations of interest (Lazar et al., 2008; Jensen et al., 2014; Glenn et al., 2015a, 2016; Henriques et al., 2015).
Contrary to DTI, higher-order diffusion models, including DKI, require multi-shell High Angular Resolution Diffusion Imaging (HARDI) sequences (Descoteaux, 1999), typically involving several high b values distributed on a high number of gradient directions, grouped in shells. This implies longer acquisition times, straining the feasibility of advanced dMRI methods of such kind in certain clinical environments due to time constraints (typical scan times are in the order of 20 min). Nevertheless, research efforts are focused on making this technique feasible within routine protocol since it has turned out to be extremely beneficial in studying microstructural changes in several preclinical and clinical research populations.
As the acquisition protocol used to obtain a DKI dataset includes all the information necessary to derive a standard DTI dataset, it can be used to calculate both types of indices. DKI measures include the Kurtosis Fractional Anisotropy (KFA) (Glenn et al., 2015b), Mean, Axial and Radial kurtosis (MK, AK, RK, respectively) (Jensen and Helpern, 2010). These measures quantify the degree of non-Gaussianity and can be regarded as indices of tissue compartmentalization or complexity. In analogy to the definition of MD, MK is defined as the average of directional kurtosis coefficients across all spatial directions, traditionally considered an index of overall microstructural complexity and compartimentalization. For voxels containing well-aligned structures, RK is defined as the average of the directional kurtosis across all directions perpendicular to the main direction of fibers. It stands for microstructural complexity along the

perpendicular diffusion direction of fibers. Conversely, AK is defined as the directional kurtosis along the main direction of well-aligned structures: it reflects microstructural complexity along the main diffusion direction. Finally, analogs to the FA of the diffusion tensor, KFA quantifies lower to higher kurtosis tensor anisotropy in a range between 0 and 1.

1.1.2 Neurite Orientation Dispersion and Density imaging

NODDI (Zhang et al., 2012) is currently the most popular among biophysical models. It provides a sufficiently simple, yet complex enough model of diffusion MRI for estimating the key features of neurite morphology in vivo on clinical MRI scanners. This model facilitates the mapping of neurite morphology and focuses on quantifying the altered architecture of these multifaceted structures. The proposed technique enables such mapping by combining a three-compartment tissue model with a two-shell HARDI protocol optimized for clinical feasibility. Specifically, it separates the signal arising from the intra-cellular compartment, extra-cellular compartment and CSF. Each of them affects water diffusion within the environment uniquely and gives rise to a separate normalized MR signal.
More in detail, the intra-cellular compartment refers to the space bounded by the membrane of neurites. This space is modeled as a set of sticks, i.e., cylinders of zero radius, to capture the highly restricted nature of diffusion perpendicular to neurites and unhindered diffusion along the axon. The orientation distribution of sticks can range from highly parallel to highly dispersed. The extra-cellular compartment refers to the space around the neurites, which is occupied by various types of glial cells and, additionally in GM, cell bodies. In this space, the diffusion of water molecules is hindered by the presence of neurites but not restricted, hence is modeled with Gaussian anisotropic diffusion. The CSF compartment models the space occupied by cerebrospinal fluid and is modeled as isotropic Gaussian diffusion.
Furthermore, to characterize the microstructure within a voxel, NODDI uses three scalar parameters: (i) Neurite Density Index (NDI), also called Intra Cellular Volume Fraction (ICVF), estimating the density of neurites; (ii) Orientation Dispersion Index (ODI), defined to characterize angular variation of neurites, reflecting the spatial configuration of the neurite structures; and (iii) free water fraction or Isotropic Volume Fraction (ISOVF), quantifying freely diffusing water from neural tissues with Gaussian diffusion.
The resulting indices of neurites have proven to relate more directly to and provide more specific markers of brain tissue microstructure than standard indices from DTI, such as FA. Specifically, NODDI provides sensible neurite density and orientation dispersion estimates, thereby disentangling two key contributing factors to FA and enabling the analysis of each factor individually. The optimized protocol takes about 30 min to acquire, making it feasible for inclusion in a typical clinical setting. Nevertheless, sampling fewer orientations in each shell can reduce the acquisition time to just 10 min with minimal impact on the accuracy of the estimates. This demonstrates the feasibility of NODDI even for the most time-sensitive clinical applications, such as neonatal and dementia imaging. By increasing specificity for certain clinically meaningful tissue properties, the application of NODDI in clinical research has mostly reported promising results for improving patient stratification and prediction of neurological functions. In particular, given the key role neurite morphology covers in terms of brain development, NODDI has opened new opportunities for understanding neurodevelopment and disorders.

1.1.3 Multi-Shell Multi-Tissue Constrained Spherical Deconvolution

MSMT CSD (Jeurissen et al., 2014) is a specific extension of the Spherical Deconvolution (SD) approach. SD is a particularly attractive HARDI method, which provides estimates of the full fODF in each brain voxel, regardless of the number of underlying fiber orientations (Tournier et al., 2004). It is based on the assumption that the dMRI signal originating from a single voxel (made up of different fiber populations) is

given by the spherical convolution of the single response function (the dMRI signal profile for a typical fiber population) with the fODF (the apparent density of fibers as a function of orientation). The unknown represented by the fODF can thus be found by performing the deconvolution of the response function from the measured dMRI signal.
However, SD operation is inherently ill-posed and susceptible to noise. To sort this out, CSD introduces a constraint to minimize the appearance of physically impossible negative values in the reconstructed fODF. With this constraint, it becomes possible to perform the SD operation with drastically reduced noise sensitivity, allowing reliable fODF estimates on clinically feasible dMRI data (Tournier et al., 2007).
Despite the improvements provided by CSD, significant challenges persist. First, CSD typically only supports data acquired with a single shell acquisition scheme -i.e., single constant diffusion weighting, despite multi-shell data becoming more and more prevalent in microstructural modelling. Furthermore, CSD can only provide high quality fODF estimates in voxels containing WM only. Conversely, in voxels including other tissue types such as GM and CSF, the WM response function may no longer be appropriate and SD produces unreliable, noisy fODF estimates.
To this end, MSMT CSD approach is conceived as an extension of CSD to support multi-shell dMRI data. Indeed, by exploiting the unique b value dependencies of the different tissue types, it can estimate a multi-tissue ODF. Moreover, as MSMT-CSD includes separate compartments for each tissue type, it can produce a map of the WM/GM/CSF volume fractions directly from the dMRI data, which can serve as new quantitative metrics. In addition, the more complete modeling of the dMRI signal results in more precise fiber orientation estimates at the tissue interfaces, resulting in more accurate fiber tracking in large parts of the brain compared to standard Single-Shell Single-Tissue CSD.

1.1.4 Fiber ORientation Estimated using Continuous Axially Symmetric Tensors

FORECAST (Anderson, 2005; Kaden et al., 2016) is a simple, axially symmetric model of diffusion in WM fibers used to relate diffusion measurements to fiber properties. This method represents a good trade-off between a conventional, single tensor model of diffusion and ”model-free” high angular resolution methods.
This approach is based on the invariance property of diffusion signal: as long as the local structure is axially symmetric - which is typically the case in nerve tissue - for a fixed b value the spherical mean of the diffusion signal over the gradient directions does not depend on the axon orientation distribution. This allows the disentanglement of intrinsic fiber diffusivity from fiber orientation distribution, enabling the extraction of relevant markers of axon microgeometry that are unaffected by fiber dispersion and crossing, which are ubiquitous in brain white matter.. This translates into potentially improving the sensitivity and/or specificity to various neurological conditions.
FORECAST models the single fiber response within each voxel with an axially symmetric tensor and exploits the mean of the signal to estimate the tensor parameters. Its associated scalar indices are the crossing invariant version of tensor indices: the parallel diffusivity (d_par), the perpendicular diffusivity (d_perp), the fractional anisotropy (FORECAST-fa) and the mean diffusivity (md) in each voxel.
These quantitative maps of the voxel-averaged diffusion coefficients can be estimated in a clinically feasible manner in vivo. Indeed, this method does not assume prior knowledge about fibers' orientation inside a voxel, neither normally requires complex gradient waveforms with multiple gradient pulses. It is instead able to recover microscopic diffusion anisotropy resorting to a widely available pulse sequence featuring moderate levels of diffusion weighting, which simplifies implementation and improves image quality compared to very high diffusion weighting methods.
Factoring out the effects due to fiber dispersion and crossing in human WM, this technique has proven

to resolve crossing fibers better than other existing methods and also to address the problem of partial volume averaging in DTI, providing a basis for more reliable estimates of fiber orientation and anisotropy. In contrast to DTI, it can discriminate changes in angular distribution (”coherence”) from changes in fiber anisotropy, which potentially improves the sensitivity and/or specificity of image-derived parameters for diseases that directly or indirectly affect WM.

1.2 Approaches

1.2.1 Tract-Based Spatial Statistics

Falling within the sphere of inference, TBSS is a theoretical, knowledge-driven statistic aimed at unraveling the biological underpinnings of the observed question (e.g., pathological condition). Specifically, it is an automated, observer-independent approach for assessing DTI Fractional Anisotropy (DTI-FA) in major WM tracts on a voxel-wise basis across groups of subjects (Smith et al., 2006). This method has been used extensively on scans acquired at Term-Equivalent-Age (TEA) in preterm-born neonates to successfully detect alterations in WM microstructure in the absence of overt brain injury and to predict cognitive and motor outcomes in young preterm-born children (Tortora et al., 2018; Duerden et al., 2015; Counsell et al., 2002), which is highly relevant to clinicians making essential care decisions. The reasons behind the popularity of TBSS reside in being an objective, sensitive, and relatively easy-to-interpret method for multi-subject, whole-brain diffusion data analysis. This allows overcoming some of the limitations of a common ROI-based approach to analyze neonatal data, including subjectivity, manual intensity, intra- and inter-subject variability, and a priori spatial localization, (Ly et al., 2015). Although it remains the leading technique for voxel-wise DTI analysis, the application of TBSS is not without its pitfalls. These include the influence of noise levels, parameter settings, and the choice of template, quality of image registration on the resulting anatomical specificity, low sensitivity for detecting subtle wide-spread abnormalities, and impossibility to develop individual-based imaging indices. All of these factors in turn question the reproducibility and robustness of the final TBSS result, which is essential for establishing biomarkers and diagnostic/prognostic indices at the individual level (Bach et al., 2014).

1.2.2 Support Vector Machine

In this respect, the advent of predictive approaches in the early 2000s has revolutionized neuroimaging studies. Compared to inference-based analysis, predictive models can extract unbiased, individualized biomarkers of diseases or functional brain states of fundamental importance in diagnosis, prognosis, and patient stratification (Davatzikos, 2019). Previous studies have focused on SVM (Golland et al., 2002; Lao et al., 2004), which has been a cornerstone in this field, largely because of its robustness and ease of use with a variety of kernels (Schölkopf et al., 2002). SVM is a prediction-oriented, data-driven methodology representing a good trade-off between model transparency for easy interpretability and understanding and model complexity for capturing complex interrelationships, which makes it ideal for the clinical research domain, unlike many complex nonlinear predictive models. These and other methods have already been widely applied in neuroimaging studies regarding preterm birth (Chu et al., 2015; Galdi et al., 2020; Saha et al., 2020), with the vast majority of works resorting to TBSS as a preprocessing step preparatory to the application of Machine Learning (ML) predictive algorithms.
However, to the best of our knowledge, this is the first study using univariate statistics and predictive modeling jointly on intramodal dMRI to explore the most discriminating WM regions representative of such a current phenomenon as preterm birth. Indeed, while the two methods have traditionally been employed separately, resorting to the two modeling goals in conjunction allows us to strengthen our findings about

the identification of robust markers for WM alterations associated with preterm birth. More broadly, a more thorough understanding of the different ways to define important associations in the observed data is key to a patient-centered, personalized medical approach.

1.2.3 Canonical correlation Analysis

CCA (Hardoon et al., 2004), focusing on finding linear combinations that account for the most correlation in two or more datasets, has proved to outperform traditional statistical tools in unlocking the complex relationships among many variables in large datasets (Wang et al., 2020). Different dMRI metrics themselves share overlying information, potentially causing partial redundancies in data analysis, especially in the case of neonatal imaging due to refinement of tract diffusion properties in development (Bzdok et al., 2020; Chamberland et al., 2019; Cox et al., 2016; De Santis et al., 2014; Girault et al., 2019). This, in turn, hints that the identification of a reduced number of microstructurally informative and biologically-interpretable components may turn out to be particularly useful. Relating to our survey, CCA is specifically beneficial for capitalizing on the strength of each microstructural imaging feature to uncover hidden relationships among separate microstructural models. Despite its multiple applications in medical imaging (Correa et al., 2008, 2010a,b; Hardoon et al., 2007; Sui et al., 2011, 2013) as far as we are aware, no study has used intramodal advanced dMRI data fusion to examine the full relationships among multiple dMRI models to provide more informative insights into altered brain patterns typical of prematurity.

Specifically, CCA aims at decomposing each input feature into a set of Canonical Variates A_k and the corresponding Canonical Weigths W_k, given by:

$$A_k^T = W_k X_k^T \tag{S1}$$

Where: $X_k \in R^{NxV_k}, A_k \in R^{NxD}, W_k \in R^{V_kxD}$, with V_k being the number of variables in X_k, N the number of observations in X_k, and D the number of CV. From CCA, within an intramodal fusion approach, one can go backwards decomposing each input feature X_k into a set of components C_k and corresponding modulation profiles (inter-subject variations) A_k, so that:

$$X_k = A_k C_k \tag{S2}$$

with:

$$C_k = A_k^{\dagger} X_k \tag{S3}$$

In our specific case, $N = 69$ (number of subjects), $V_k = 2286$ (number of features/voxels), $D = 4$ (number of Canonical Variates), while $K = 14$ (number of microstructural measures under analysis).

2 SUPPLEMENTARY TABLES AND FIGURES

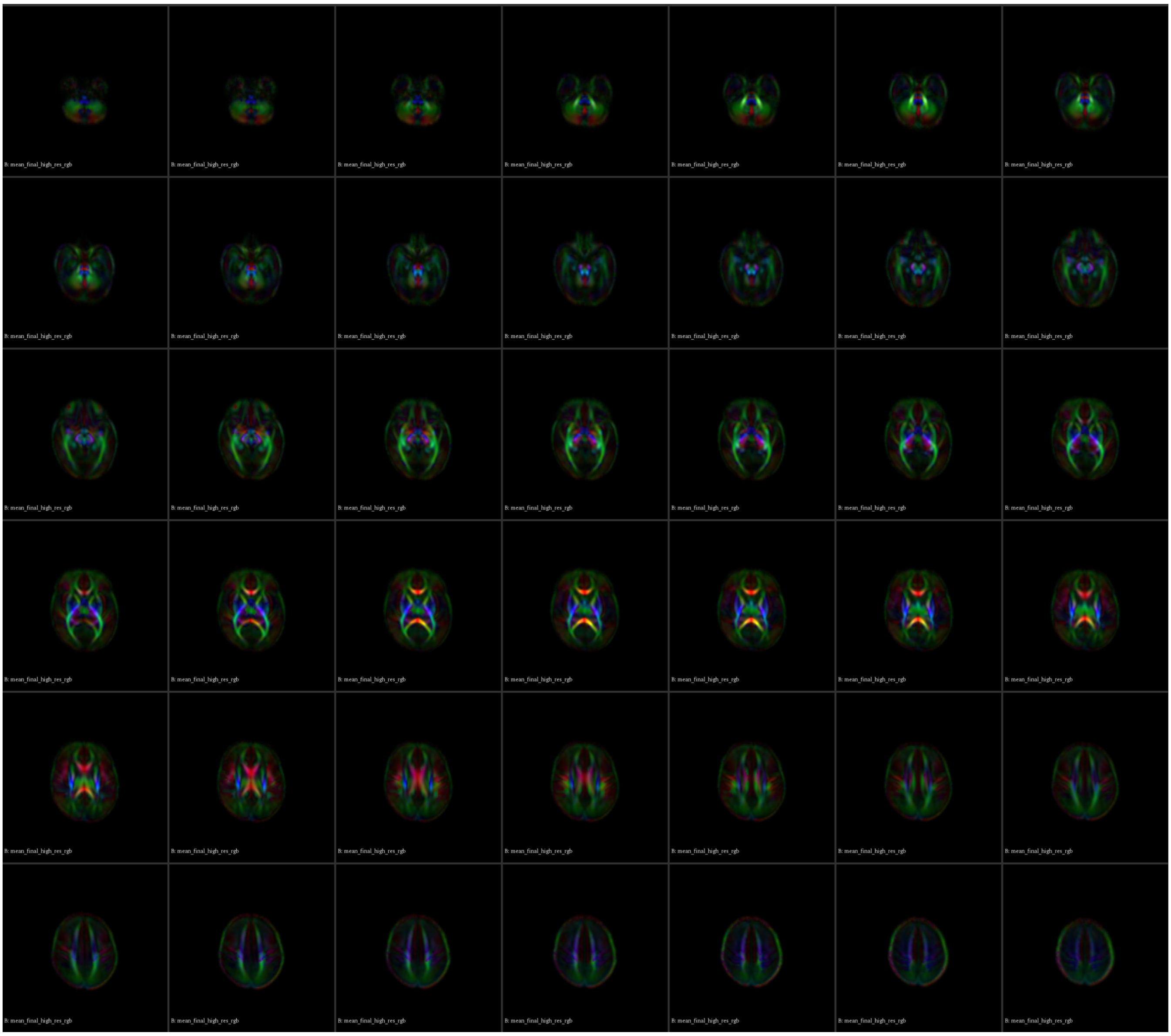

Figure S1. Population-specific DTI template: lightbox displaying axial views of the age-specific template created ad-hoc for performing normalization of DTI volumes within DTI-TK and subsequent creation of WM skeleton within TBSS.

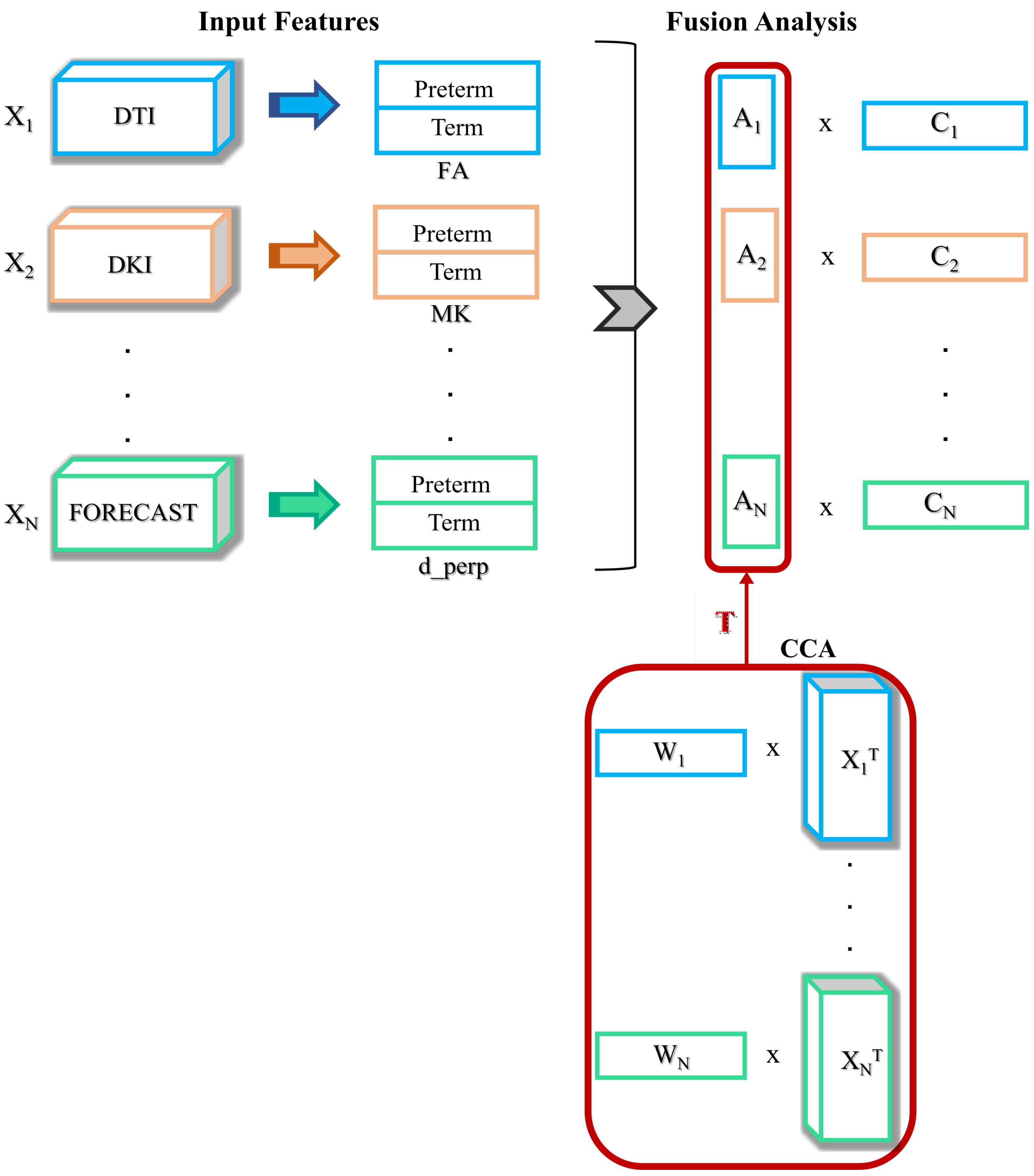

Figure S2. Canonical Correlation Analysis framework applied to our intramodal dataset on all 14 HARDI microstructural measures.

REFERENCES

Anderson, A. W. (2005). Measurement of fiber orientation distributions using high angular resolution diffusion imaging. *Magnetic Resonance in Medicine: An Official Journal of the International Society for Magnetic Resonance in Medicine* 54, 1194–1206

Assaf, Y. and Basser, P. J. (2005). Composite hindered and restricted model of diffusion (charmed) mr imaging of the human brain. *Neuroimage* 27, 48–58

Bach, M., Laun, F. B., Leemans, A., Tax, C. M., Biessels, G. J., Stieltjes, B., et al. (2014). Methodological considerations on tract-based spatial statistics (tbss). *Neuroimage* 100, 358–369

Bzdok, D., Engemann, D., and Thirion, B. (2020). Inference and prediction diverge in biomedicine. *Patterns* 1, 100119

Chamberland, M., Raven, E. P., Genc, S., Duffy, K., Descoteaux, M., Parker, G. D., et al. (2019). Dimensionality reduction of diffusion mri measures for improved tractometry of the human brain. *NeuroImage* 200, 89–100

Chu, C., Lagercrantz, H., Forssberg, H., and Nagy, Z. (2015). Investigating the use of support vector machine classification on structural brain images of preterm–born teenagers as a biological marker. *Plos one* 10, e0123108

Correa, N. M., Adali, T., Li, Y.-O., and Calhoun, V. D. (2010a). Canonical correlation analysis for data fusion and group inferences. *IEEE signal processing magazine* 27, 39–50

Correa, N. M., Eichele, T., Adalı, T., Li, Y.-O., and Calhoun, V. D. (2010b). Multi-set canonical correlation analysis for the fusion of concurrent single trial erp and functional mri. *Neuroimage* 50, 1438–1445

Correa, N. M., Li, Y.-O., Adali, T., and Calhoun, V. D. (2008). Canonical correlation analysis for feature-based fusion of biomedical imaging modalities and its application to detection of associative networks in schizophrenia. *IEEE journal of selected topics in signal processing* 2, 998–1007

Counsell, S. J., Maalouf, E. F., Fletcher, A. M., Duggan, P., Battin, M., Lewis, H. J., et al. (2002). Mr imaging assessment of myelination in the very preterm brain. *American journal of neuroradiology* 23, 872–881

Cox, S. R., Ritchie, S. J., Tucker-Drob, E. M., Liewald, D. C., Hagenaars, S. P., Davies, G., et al. (2016). Ageing and brain white matter structure in 3,513 uk biobank participants. *Nature communications* 7, 1–13

Davatzikos, C. (2019). Machine learning in neuroimaging: Progress and challenges. *Neuroimage* 197, 652

De Santis, S., Drakesmith, M., Bells, S., Assaf, Y., and Jones, D. K. (2014). Why diffusion tensor mri does well only some of the time: variance and covariance of white matter tissue microstructure attributes in the living human brain. *Neuroimage* 89, 35–44

Descoteaux, M. (1999). High angular resolution diffusion imaging (hardi). *Wiley encyclopedia of electrical and electronics engineering* , 1–25

Duerden, E., Foong, J., Chau, V., Branson, H., Poskitt, K., Grunau, R., et al. (2015). Tract-based spatial statistics in preterm-born neonates predicts cognitive and motor outcomes at 18 months. *American Journal of Neuroradiology* 36, 1565–1571

Fieremans, E., Benitez, A., Jensen, J., Falangola, M., Tabesh, A., Deardorff, R., et al. (2013). Novel white matter tract integrity metrics sensitive to alzheimer disease progression. *American Journal of Neuroradiology* 34, 2105–2112

Fieremans, E., Jensen, J. H., and Helpern, J. A. (2011). White matter characterization with diffusional kurtosis imaging. *Neuroimage* 58, 177–188

Galdi, P., Blesa, M., Stoye, D. Q., Sullivan, G., Lamb, G. J., Quigley, A. J., et al. (2020). Neonatal morphometric similarity mapping for predicting brain age and characterizing neuroanatomic variation associated with preterm birth. *NeuroImage: Clinical* 25, 102195

Girault, J. B., Munsell, B. C., Puechmaille, D., Goldman, B. D., Prieto, J. C., Styner, M., et al. (2019). White matter connectomes at birth accurately predict cognitive abilities at age 2. *Neuroimage* 192, 145–155

Glenn, G. R., Helpern, J. A., Tabesh, A., and Jensen, J. H. (2015a). Optimization of white matter fiber tractography with diffusional kurtosis imaging. *NMR in Biomedicine* 28, 1245–1256

Glenn, G. R., Helpern, J. A., Tabesh, A., and Jensen, J. H. (2015b). Quantitative assessment of diffusional kurtosis anisotropy. *NMR in Biomedicine* 28, 448–459

Glenn, G. R., Kuo, L.-W., Chao, Y.-P., Lee, C.-Y., Helpern, J. A., and Jensen, J. H. (2016). Mapping the orientation of white matter fiber bundles: a comparative study of diffusion tensor imaging, diffusional kurtosis imaging, and diffusion spectrum imaging. *American Journal of Neuroradiology* 37, 1216–1222

Golland, P., Fischl, B., Spiridon, M., Kanwisher, N., Buckner, R. L., Shenton, M. E., et al. (2002). Discriminative analysis for image-based studies. In *International Conference on Medical Image Computing and Computer-Assisted Intervention* (Springer), 508–515

Grossman, E. J., Ge, Y., Jensen, J. H., Babb, J. S., Miles, L., Reaume, J., et al. (2012). Thalamus and cognitive impairment in mild traumatic brain injury: a diffusional kurtosis imaging study. *Journal of neurotrauma* 29, 2318–2327

Hardoon, D. R., Mourao-Miranda, J., Brammer, M., and Shawe-Taylor, J. (2007). Unsupervised analysis of fmri data using kernel canonical correlation. *NeuroImage* 37, 1250–1259

Hardoon, D. R., Szedmak, S., and Shawe-Taylor, J. (2004). Canonical correlation analysis: An overview with application to learning methods. *Neural computation* 16, 2639–2664

Henriques, R. N., Correia, M. M., Nunes, R. G., and Ferreira, H. A. (2015). Exploring the 3d geometry of the diffusion kurtosis tensor—impact on the development of robust tractography procedures and novel biomarkers. *Neuroimage* 111, 85–99

Henriques, R. N., Jespersen, S. N., and Shemesh, N. (2019). Microscopic anisotropy misestimation in spherical-mean single diffusion encoding mri. *Magnetic resonance in medicine* 81, 3245–3261

Huber, E., Henriques, R. N., Owen, J. P., Rokem, A., and Yeatman, J. D. (2019). Applying microstructural models to understand the role of white matter in cognitive development. *Developmental Cognitive Neuroscience* 36, 100624

Hui, E. S., Fieremans, E., Jensen, J. H., Tabesh, A., Feng, W., Bonilha, L., et al. (2012). Stroke assessment with diffusional kurtosis imaging. *Stroke* 43, 2968–2973

Jensen, J. H. and Helpern, J. A. (2010). Mri quantification of non-gaussian water diffusion by kurtosis analysis. *NMR in Biomedicine* 23, 698–710

Jensen, J. H., Helpern, J. A., Ramani, A., Lu, H., and Kaczynski, K. (2005). Diffusional kurtosis imaging: the quantification of non-gaussian water diffusion by means of magnetic resonance imaging. *Magnetic Resonance in Medicine: An Official Journal of the International Society for Magnetic Resonance in Medicine* 53, 1432–1440

Jensen, J. H., Helpern, J. A., and Tabesh, A. (2014). Leading non-gaussian corrections for diffusion orientation distribution function. *NMR in Biomedicine* 27, 202–211

Jespersen, S. N., Kroenke, C. D., Østergaard, L., Ackerman, J. J., and Yablonskiy, D. A. (2007). Modeling dendrite density from magnetic resonance diffusion measurements. *Neuroimage* 34, 1473–1486

Jeurissen, B., Tournier, J.-D., Dhollander, T., Connelly, A., and Sijbers, J. (2014). Multi-tissue constrained spherical deconvolution for improved analysis of multi-shell diffusion mri data. *NeuroImage* 103, 411–426

Kaden, E., Kruggel, F., and Alexander, D. C. (2016). Quantitative mapping of the per-axon diffusion coefficients in brain white matter. *Magnetic resonance in medicine* 75, 1752–1763

Lampinen, B., Szczepankiewicz, F., Mårtensson, J., van Westen, D., Sundgren, P. C., and Nilsson, M. (2017). Neurite density imaging versus imaging of microscopic anisotropy in diffusion mri: a model comparison using spherical tensor encoding. *Neuroimage* 147, 517–531

Lao, Z., Shen, D., Xue, Z., Karacali, B., Resnick, S. M., and Davatzikos, C. (2004). Morphological classification of brains via high-dimensional shape transformations and machine learning methods. *Neuroimage* 21, 46–57

Lazar, M., Jensen, J. H., Xuan, L., and Helpern, J. A. (2008). Estimation of the orientation distribution function from diffusional kurtosis imaging. *Magnetic Resonance in Medicine: An Official Journal of the International Society for Magnetic Resonance in Medicine* 60, 774–781

Lin, L., Bhawana, R., Xue, Y., Duan, Q., Jiang, R., Chen, H., et al. (2018). Comparative analysis of diffusional kurtosis imaging, diffusion tensor imaging, and diffusion-weighted imaging in grading and assessing cellular proliferation of meningiomas. *American Journal of Neuroradiology* 39, 1032–1038

Ly, M. T., Nanavati, T. U., Frum, C. A., and Pergami, P. (2015). Comparing tract-based spatial statistics and manual region-of-interest labeling as diffusion analysis methods to detect white matter abnormalities in infants with hypoxic-ischemic encephalopathy. *Journal of Magnetic Resonance Imaging* 42, 1689–1697

Marrale, M., Collura, G., Brai, M., Toschi, N., Midiri, F., La Tona, G., et al. (2016). Physics, techniques and review of neuroradiological applications of diffusion kurtosis imaging (dki). *Clinical neuroradiology* 26, 391–403

Nilsson, M., Lätt, J., Ståhlberg, F., van Westen, D., and Hagslätt, H. (2012). The importance of axonal undulation in diffusion mr measurements: a monte carlo simulation study. *NMR in Biomedicine* 25, 795–805

Novikov, D. S., Kiselev, V. G., and Jespersen, S. N. (2018). On modeling. *Magnetic resonance in medicine* 79, 3172–3193

Rudrapatna, S. U., Wieloch, T., Beirup, K., Ruscher, K., Mol, W., Yanev, P., et al. (2014). Can diffusion kurtosis imaging improve the sensitivity and specificity of detecting microstructural alterations in brain tissue chronically after experimental stroke? comparisons with diffusion tensor imaging and histology. *Neuroimage* 97, 363–373

Saha, S., Pagnozzi, A., Bourgeat, P., George, J. M., Bradford, D., Colditz, P. B., et al. (2020). Predicting motor outcome in preterm infants from very early brain diffusion mri using a deep learning convolutional neural network (cnn) model. *Neuroimage* 215, 116807

Schölkopf, B., Smola, A. J., Bach, F., et al. (2002). *Learning with kernels: support vector machines, regularization, optimization, and beyond* (MIT press)

Smith, S. M., Jenkinson, M., Johansen-Berg, H., Rueckert, D., Nichols, T. E., Mackay, C. E., et al. (2006). Tract-based spatial statistics: voxelwise analysis of multi-subject diffusion data. *Neuroimage* 31, 1487–1505

Steven, A. J., Zhuo, J., and Melhem, E. R. (2014). Diffusion kurtosis imaging: an emerging technique for evaluating the microstructural environment of the brain. *American journal of roentgenology* 202, W26–W33

Sui, J., He, H., Yu, Q., Chen, J., Rogers, J., Pearlson, G. D., et al. (2013). Combination of resting state fmri, dti, and smri data to discriminate schizophrenia by n-way mcca+ jica. *Frontiers in human neuroscience* 7, 235

Sui, J., Pearlson, G., Caprihan, A., Adali, T., Kiehl, K. A., Liu, J., et al. (2011). Discriminating schizophrenia and bipolar disorder by fusing fmri and dti in a multimodal cca+ joint ica model. *Neuroimage* 57, 839–855

Tortora, D., Martinetti, C., Severino, M., Uccella, S., Malova, M., Parodi, A., et al. (2018). The effects of mild germinal matrix-intraventricular haemorrhage on the developmental white matter microstructure of preterm neonates: a dti study. *European radiology* 28, 1157–1166

Tournier, J.-D., Calamante, F., and Connelly, A. (2007). Robust determination of the fibre orientation distribution in diffusion mri: non-negativity constrained super-resolved spherical deconvolution. *Neuroimage* 35, 1459–1472

Tournier, J.-D., Calamante, F., Gadian, D. G., and Connelly, A. (2004). Direct estimation of the fiber orientation density function from diffusion-weighted mri data using spherical deconvolution. *Neuroimage* 23, 1176–1185

Wang, H.-T., Smallwood, J., Mourao-Miranda, J., Xia, C. H., Satterthwaite, T. D., Bassett, D. S., et al. (2020). Finding the needle in a high-dimensional haystack: Canonical correlation analysis for neuroscientists. *NeuroImage* 216, 116745

Zhang, H., Schneider, T., Wheeler-Kingshott, C. A., and Alexander, D. C. (2012). Noddi: practical in vivo neurite orientation dispersion and density imaging of the human brain. *Neuroimage* 61, 1000–1016

Zhu, T., Peng, Q., Ouyang, A., and Huang, H. (2021). Neuroanatomical underpinning of diffusion kurtosis measurements in the cerebral cortex of healthy macaque brains. *Magnetic Resonance in Medicine* 85, 1895–1908
